# Targeting RBM10‐Repressed RORB Activity in Liquid Condensates Inhibits Lysosomal Biogenesis and Neuroblastoma Progression via Affecting NF‐κB Signaling

**DOI:** 10.1002/advs.202506131

**Published:** 2025-09-03

**Authors:** Yanhua Guo, Xiaojing Wang, Chunhui Yang, Zhijie Wang, Xiaolin Wang, Xinyue Li, Jiaying Qu, Shunchen Zhou, Liduan Zheng, Qiangsong Tong

**Affiliations:** ^1^ Department of Pediatric Surgery, Union Hospital, Tongji Medical College Huazhong University of Science and Technology 1277 Jiefang Avenue Wuhan Hubei Province 430022 P. R. China; ^2^ Department of Pathology, Union Hospital, Tongji Medical College Huazhong University of Science and Technology 1277 Jiefang Avenue Wuhan Hubei Province 430022 P. R. China; ^3^ Department of Geriatrics, Union Hospital, Tongji Medical College Huazhong University of Science and Technology 1277 Jiefang Avenue Wuhan Hubei Province 430022 P. R. China

**Keywords:** lysosomal biogenesis, neuroblastoma, nuclear factor kappa B, RAR‐related orphan receptor B, RNA binding motif protein 10

## Abstract

Neuroblastoma (NB), a pediatric solid malignancy, is distinguished by hetergenous clinical characteristics, including tumor aggressiveness or spontaneous regression. Nevertheless, the regulatory mechanisms and therapeutic approaches underlying these processes are still mainly unknown. Herein, RAR‐related orphan receptor B (RORB) as a transcription factor repressing nuclear factor kappa B (NF‐κB) signaling involved in lysosomal biogenesis of NB. *RORB* attenuated the growth, invasiveness, and metastatic spread of NB cells are identified. From a mechanistic perspective, *RORB* increased the transcription of nuclear receptor subfamily 1 group D member 1 (*NR1D1*)*‐* or RIO kinase 3 (*RIOK3*)*‐* in a circadian clock‐dependent manner, resulting in suppression of NF‐κB activity, subsequent derepression of folliculin (*FLCN*)*‐* or folliculin interacting protein 1 (*FNIP1*)*‐* levels, and decrease of lysosomal biogenesis in NB cells. Meanwhile, in liquid condensates, RNA binding motif protein 10 (RBM10) interacted with RORB to repress its transactivation and exerted oncogenic roles in lysosomal biogenesis and aggressiveness of NB cells. Pre‐clinically, a small peptide is able to block the interaction between RBM10 and RORB, and suppresses lysosomal biogenesis, tumorigenesis, and aggressiveness. High levels of *RORB*, *NR1D1*, *RIOK3*, *FLCN*, and *FNIP1*, or low expression of *RBM10*, are linked to favorable prognosis of clinical NB cases. These results indicate that targeting RBM10‐repressed RORB activity in liquid condensates inhibits lysosomal biogenesis and NB progression via affecting NF‐κB signaling.

## Graphical abstract

This study identifies that RBM10 represses the transcriptional activity of RORB in liquid condensates, resulting in down‐regulation of *NR1D1* and *RIOK3* that further activates NF‐κB activity to facilitate lysosomal biogenesis and aggressive features of NB cells. A peptide blocking RBM10‐RORB interaction suppresses the progression of NB, highlighting the *RBM10*/*RORB*/*NF‐κB* axis as a novel therapeutic target for tumors.

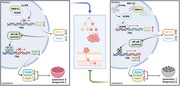

## 1 Introduction

Neuroblastoma (NB), a solid malignant neoplasm originating from sympathoadrenal system, is prevalent in pediatric patients under 5 years of age.^[^ ^1^ ^]^ Tumor invasion and metastasis continue to be the primary causes of poor clinical outcome in individuals with advanced NB.^[^ ^1^ ^]^ Interestingly, some NB patients with a special pattern of metastasis at stage 4S of International Neuroblastoma Staging System (INSS) have a surprisingly favorable outcome, especially in infants with age less than 12 months. These cases have a high likelihood of spontaneous regression or differentiation.^[^ ^1^, ^2^ ^]^ It has been established that near‐triploidy predominates in stage 1, 2, or 4S tumors and is associated with excellent prognosis, while diploidy or tetraploidy indicating poor outcome is more frequent in stage 4 NB specimens, suggesting the linkage of tumor cell ploidy to clinical outcomes of NB patients.^[^ ^3^ ^]^ Meanwhile, recent studies indicate that genomic, biological, or immunological mechanisms are associated with the aggressive behaviors or spontaneous regression of NB,^[^ ^2^ ^]^ suggesting that elucidation of mechanisms underlying different NB behaviors is crucial for proposing therapeutic approaches. However, systematic screening of transcription factors (TFs) regulating gene expression during NB progression remains elusive.

In recent years, increasing evidence reveals that disruption of circadian rhythms contributes to onset of various chronic diseases, including cancers.^[^ ^4^ ^]^ For example, brain and muscle ARNT‐like 1 (*BMAL1*) suppresses the biological features of NB cells, and is linked to a favorable prognosis of patients.^[^ ^5^ ^]^ By controlling gene transcription necessary for homologous recombination, cryptochrome circadian regulator 1 (*CRY1*) promotes DNA repair and cellular survival, and correlates with worse survival outcomes in prostate cancer.^[^ ^6^ ^]^ RAR‐related orphan receptor B (*RORB*) belongs to retinoic acid‐related orphan receptor family,^[^ ^7^ ^]^ and acts as a circadian transcription factor to exert essential regulatory function in the preservation of certain physiological processes, such as bone formation and circadian rhythm.^[^ ^7^ ^]^ In colorectal cancer‐initiating cells, *RORB* serves as a transcriptional factor to modulate Wnt pathway by interacting with HMG‐box transcription factor 1.^[^ ^8^ ^]^ Of note, *RORB* is able to drive transcription in nuclear extracts of murine NB cell line Neuro2A, but not in that of non‐neuronal cancer cells.^[^ ^9^ ^]^ However, the functions of *RORB* and its protein partner in tumor progression remain unknown.

In current investigation, by performing a comprehensive analysis, we discover that RORB is a crucial transcription factor repressing NB progression. RORB transcriptionally activates its target genes, nuclear receptor subfamily 1 group D member 1 (*NR1D1*) and RIO kinase 3 (*RIOK3*), in a circadian clock‐dependent manner, leading to attenuated activation of nuclear factor kappa B (NF‐κB) signaling, subsequent de‐repression of folliculin (*FLCN*) and folliculin interacting protein 1 (*FNIP1*) levels, and decrease of lysosomal biogenesis in NB. Meanwhile, RNA binding motif protein 10 (RBM10) physically interacts with and represses RORB transactivation in liquid condensates to exert oncogenic roles in growth, invasiveness, and metastatic spread of NB. Pre‐clinically, treatment with inhibitory peptide blocking RBM10‐RORB interaction suppresses lysosomal biogenesis and aggressive features of tumor cells, highlighting the biological significance of *RBM10*/*RORB*/*NF‐κB* axis in NB progression.

## 2 Results

### 2.1 Transcription Factor RORB Inhibits the Progression of NB

**Figure 1:**
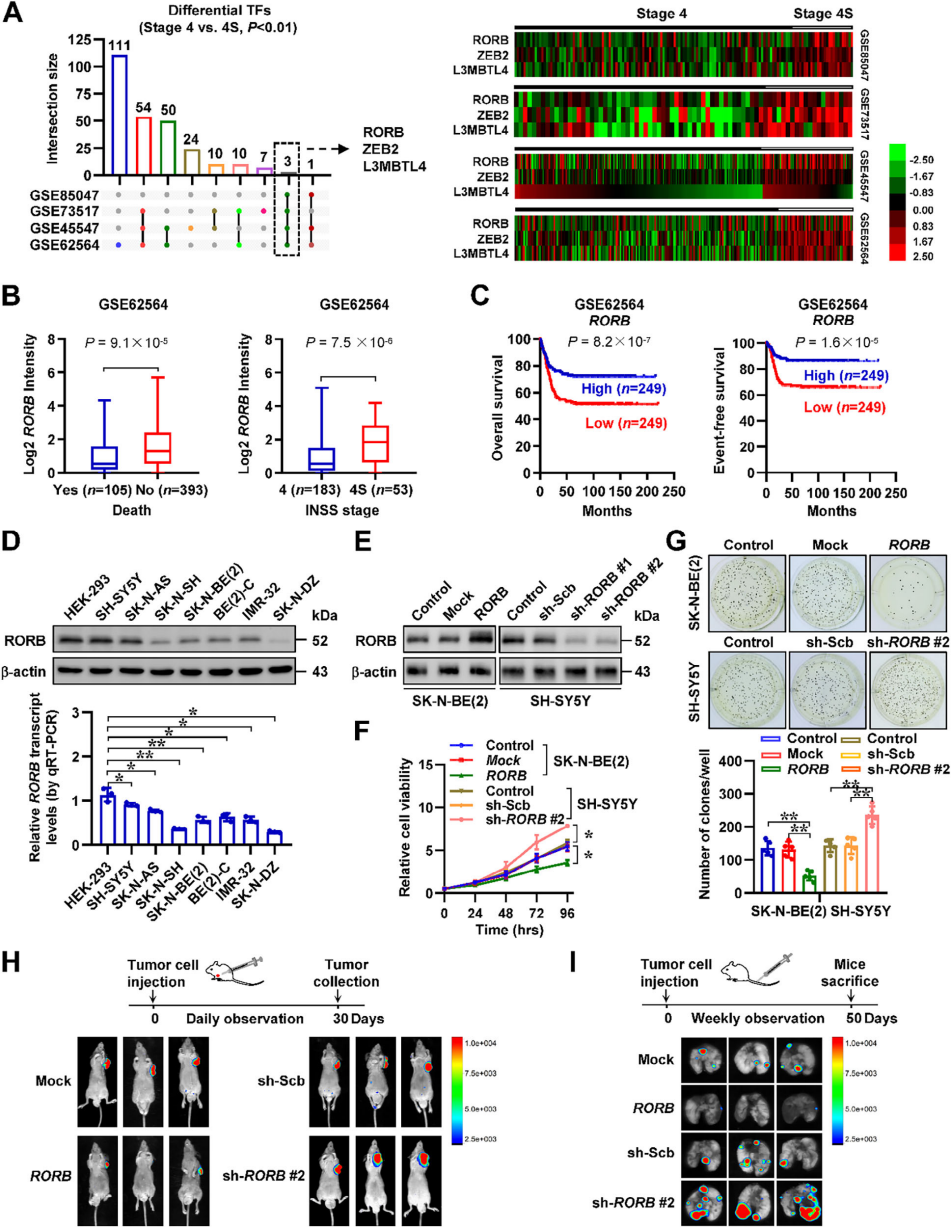
Transcription factor RORB inhibits the progression of NB. A) Upset diagram (left panel) and heatmap (right panel) showing the identification and profiling of differentially expressed transcription factors (TFs) in 498 (GSE62564), 649 (GSE45547), 105 (GSE73517), and 283 (GSE85047) NB cases with varied INSS stages (4S versus 4, *p* < 0.01). B) Mining of a public RNA‐seq dataset (GSE62564) revealing the *RORB* levels in 498 NB tissues with different status of death or INSS stages. C) Kaplan‐Meier curve showing overall or event‐free survival of 498 NB patients (GSE62564) with high or low *RORB* expression (cutoff values = 2.12 and 1.44). D) Western blot (upper panel) and real‐time qRT‐PCR (lower panel, normalized to *β‐actin*, *n* = 3) assays showing the protein and transcript levels of *RORB* in NB cell lines and HEK‐293 cells. E) Western blot assay indicating the expression of RORB in untreated (control) SK‐N‐BE(2) and SH‐SY5Y parental cells, and those stably transfected with empty vector (mock), *RORB*, scramble shRNA (sh‐Scb), sh‐*RORB* #1, or sh‐*RORB* #2. F) MTT colorimetric assay indicating the viability of untreated (control) SK‐N‐BE(2) or SH‐SY5Y parental cells, and those stably transfected with mock, *RORB*, sh‐Scb, or sh‐*RORB* #2 (*n* = 4). G) Representative images (upper panel) and quantification (lower panel) of soft agar assay showing the growth of untreated (control) SK‐N‐BE(2) and SH‐SY5Y parental cells, and those stably transfected with mock, *RORB*, sh‐Scb, or sh‐*RORB* #2 (*n* = 5). H) Representative images of subcutaneous xenograft tumors formed by SK‐N‐BE(2) or SH‐SY5Y cells stably transfected with mock, *RORB*, sh‐Scb, or sh‐*RORB* #2 in nude mice (*n* = 3 per group). I) In vivo imaging of lungs in nude mice (*n* = 3 per group) treated with tail vein injection of SK‐N‐BE(2) or SH‐SY5Y stably transfected with mock, *RORB*, sh‐Scb, or sh‐*RORB* #2. Fisher's exact test for over‐lapping analysis in A. Non‐parametric Mann‐Whitney U test compared the difference in B. Log‐rank test for survival comparison in C. One‐way analysis of variance (ANOVA) or Student's *t* test compared the difference in D, F, and G. Data are shown as mean ± s.e.m. (error bars); *, *p* < 0.05; **, *p* < 0.01.

To systematically identify ‐TFs‐ regulating NB progression, we integrated and analyzed four independent public datasets derived from 498 (GSE62564), 649 (GSE45547), 105 (GSE73517), and 283 (GSE85047) NB patients. Based on over‐lapping analysis, we identified RORB, zinc finger E‐box binding homeobox 2 (ZEB2), as well as L3MBTL histone methyl‐lysine binding protein 4 (L3MBTL4) as differentially expressed TFs in these datasets with varied INSS stages (4S vs. 4, *P* < 0.01, **Figure** **1**A). Among them, only *RORB*, a circadian transcription factor, was consistently associated with favorable prognosis of NB patients in three independent NB cohorts (Table S1, Supporting Information). Notably, in 498 (GSE62564), 649 (GSE45547), and 283 (GSE85047) NB cases, *RORB* expression was reduced in tissues with death (*P* = 9.1 × 10^−5^, *P* = 2.0 × 10^−2^, *P* = 3.8 × 10^−8^) or stage 4 (*P* = 7.5 × 10^−6^, *P* = 1.2 × 10^−11^, *P* = 3.1 × 10^−7^, Figure 1B; Figure S1A, Supporting Information), which was associated with poor overall (*P* = 8.2 × 10^−7^, *P* = 1.2 × 10^−10^, *P* = 4.5 × 10^−9^) or event‐free (*P* = 1.6 × 10^−5^, *P* = 3.2 × 10^−6^, *P* = 1.0 × 10^−7^) survival of patients (Figure 1C; Figure S1B, Supporting Information). Of note, the *RORB* levels were also decreased in NB specimens with diploid status, when compared to those with near‐triploid feature (*P* = 3.7 × 10^−2^, Figure S1C, Supporting Information), and linked to favorable outcome of NB patients (*P* = 3.4 × 10^−2^) derived from TARGET database (https://ocg.cancer.gov/programs/target/projects/neuroblastoma, Figure S1C, Supporting Information). In contrast, survival analysis revealed no consistent association of circadian locomotor output cycles kaput (*CLOCK*)/*BMAL1* heterodimer levels with overall or event‐free survival in these cohorts (Figure S1D, Supporting Information). Using specific primer sets, RT‐PCR assay indicated the existence of *RORB* variant 1 (referred to *RORB*), but not of *RORB* variant 2, in NB tissues and cells (Figure S1E, Supporting Information). Western blot and real‐time quantitative RT‐PCR (qRT‐PCR) assays revealed down‐regulation of *RORB* in different multiple NB cell lines relative to HEK‐293 controls (Figure 1D). To further elucidate the roles of *RORB* in NB progression, we chose SK‐N‐BE(2), IMR‐32, SH‐SY5Y, and SK‐N‐AS (representing relatively low or high *RORB* expression) cells as models for stable over‐expression or knockdown studies, resulting in decrease or increase of cellular viability, respectively (Figure 1E,F; Figure S1F,G, Supporting Information). Stable over‐expression of *RORB* arrested cell cycle at G_0_/G_1_ phase (Figure S2A, Supporting Information), and induced slight apoptosis of SK‐N‐BE(2) and IMR‐32 cells (Figure S2B, Supporting Information), without obvious changes in expression of neuronal differentiation markers growth associated protein 43 (GAP43)^[^ ^10^ ^]^ and neurofilament 200 (NF‐200)^[^ ^10^, ^11^ ^]^ (Figure S2C, Supporting Information), when compared to untreated parental cells or those stably transfected with empty vector (mock). In addition, steady over‐expression or silencing of *RORB* reduced or promoted the anchorage‐independent growth and invasive capacity of SK‐N‐BE(2), IMR‐32, SH‐SY5Y, and SK‐N‐AS cells, than that of parental cells and those stably transfected with mock or scramble shRNA (sh‐Scb, Figure 1G; Figure S3A–C, Supporting Information). In vivo nude mice studies demonstrated that stable *RORB* over‐expression in SK‐N‐BE(2) cells significantly suppressed xenograft tumor progression, manifesting as reduced tumor volume, decreased weight, and lower Ki‐67 labeling index (Figure 1H; Figure S3D–F, Supporting Information). Moreover, reduced pulmonary dissemination and improved outcome were observed in athymic nude mice receiving tail vein injection of *RORB* over‐expressing SK‐N‐BE(2) cells (Figure 1I; Figure S3G–I, Supporting Information). Conversely, stable *RORB* knockdown in SH‐SY5Y cells significantly enhanced the growth, mass, as well as Ki‐67 labeling index of hypodermic xenograft tumors (Figure 1H; Figure S3D–F, Supporting Information), and resulted in an increase in lung metastatic nodules and significantly reduced survival of athymic nude mice (Figure 1I; Figure S3G–I, Supporting Information). These findings suggested transcription factor RORB as a potent suppressor of NB progression.

### 2.2 *RORB* Represses NF‐κB Signaling and Lysosomal Biogenesis via Up‐Regulation of *NR1D1* and *RIOK3*

**Figure 2:**
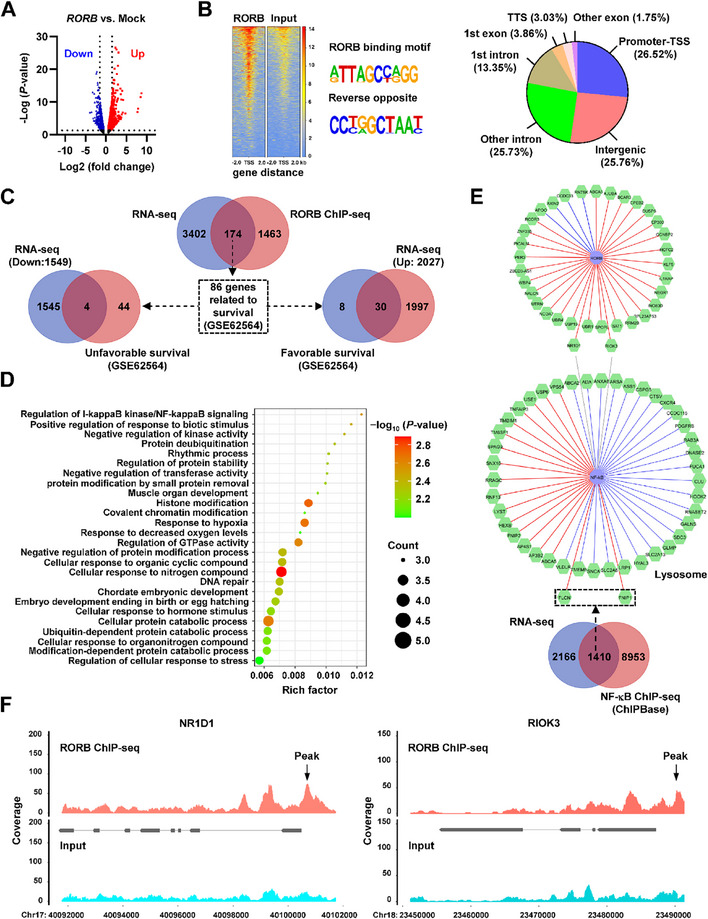
*RORB* facilitates the expression of genes regulating NF‐κB signaling pathway. A) Volcano plots of RNA‐seq assay revealing the alteration of gene expression (fold change >1.5, *p* < 0.05) in SK‐N‐BE(2) cells stably transfected with empty vector (mock) or *RORB* (*n* = 2 per group). B) Heatmap, binding motif, and distribution of ChIP‐seq assay revealing genomic enrichment of RORB in SK‐N‐BE(2) cells (*n* = 2). C) Venn diagram showing the over‐lapping analysis of 3576 altered genes in RNA‐seq assay, 1637 genes with RORB enrichment peak in ChIP‐seq assay, and their association with survival of 498 NB patients (GSE62564). D) Bubble diagram displaying the involvement of identified 34 genes in GO pathways. E) Based on analysis of RNA‐seq results and ChIPBase database (https://rnasysu.com/chipbase3), gene network revealing the expression association of RORB with 34 target genes, and that of NF‐κB with 44 lysosome‐related genes in SK‐N‐BE(2) cells with stable *RORB* over‐expression. Red and blue lines indicating up‐regulation or down‐regulation in RNA‐seq, respectively. F) ChIP‐seq assay indicating the RORB enrichment peaks on promoter regions of *NR1D1* and *RIOK3* in SK‐N‐BE(2) cells. Fisher's exact test for over‐lapping analysis in C.

**Figure 3:**
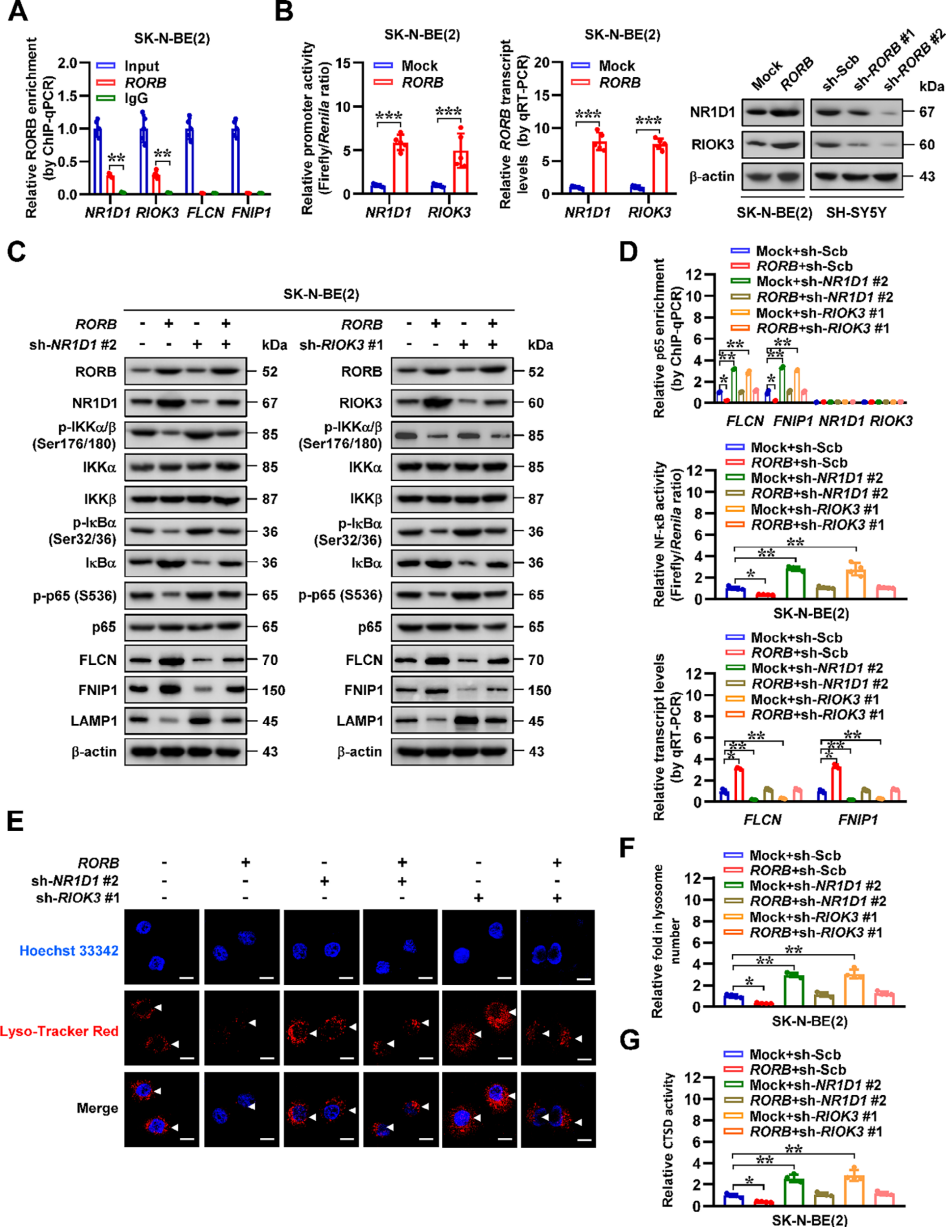
RORB inhibits NF‐κB signaling and lysosomal biogenesis via up‐regulation of *NR1D1* and *RIOK3* in NB cells. A) ChIP‐qPCR assay (normalized to input) showing the endogenous RORB enrichment on promoter regions of *NR1D1*, *RIOK3*, *FLCN*, or *FNIP1* in SK‐N‐BE(2) cells. B) Dual‐luciferase (*n* = 5), real‐time qRT‐PCR (normalized to *β‐actin*, *n* = 5), and western blot assays indicating the promoter activity, transcript, and protein levels of *NR1D1* and *RIOK3* in SK‐N‐BE(2) or SH‐SY5Y cells stably transfected with empty vector (mock), *RORB*, scramble shRNA (sh‐Scb), sh‐*RORB* #1, or sh‐*RORB* #2. C) Western blot assay revealing the levels of RORB, NR1D1, RIOK3, phosphorated or non‐phosphorated IKKα/β, IκBα, and p65, FLCN, FNIP1, and LAMP1 in SK‐N‐BE(2) cells stably transfected with mock or *RORB*, and those co‐transfected with sh‐Scb, sh*‐NR1D1* #2, or sh*‐RIOK3* #1. D) ChIP‐qPCR (normalized to input, *n* = 3), dual‐luciferase (*n* = 4), and real‐time qRT‐PCR (normalized to *β‐actin*, *n* = 4) assays indicating the enrichment or activity of p65 and transcript levels of *FLCN* or *FNIP1* in SK‐N‐BE(2) cells stably transfected with mock or *RORB*, and those co‐transfected with sh‐Scb, sh*‐NR1D1* #2, or sh*‐RIOK3* #1. E and F) Representative images E) and quantification F) of fluorescence observation showing the Lyso‐Tracker Red‐positive lysosomes (arrowheads) within SK‐N‐BE(2) cells stably transfected with mock or *RORB*, and those co‐transfected with sh‐Scb, sh*‐NR1D1* #2, or sh*‐RIOK3* #1 (*n* = 4). Scale bars: 10 µm. G) The CTSD activity in SK‐N‐BE(2) cells stably transfected with mock or *RORB*, and those co‐transfected with sh‐Scb, sh*‐NR1D1* #2, or sh*‐RIOK3* #1 (*n* = 4). Student's *t* test compared the difference in A and B. One‐way ANOVA compared the difference in D, F, and G. Data are shown as mean ± s.e.m. (error bars); *, *p* < 0.05; **, *p* < 0.01; ***, *p* < 0.001.

To further reveal *RORB* downstream targets in SK‐N‐BE(2) cells, transcriptome profiling by RNA sequencing (RNA‐seq) assay identified 2027 elevated and 1549 reduced gene transcripts (fold change>1.5, adjusted *p* < 0.05) following ectopic expression of *RORB* (**Figure** **2**A). Using RORB‐specific antibody, chromatin immunoprecipitation sequencing (ChIP‐seq) assay was performed to reveal its enrichment on genomic regions, especially on gene promoter‐transcription start sites (TSS, Figure 2B). By comprehensive analysis of 3576 altered genes in RNA‐seq assay and 1637 genes with RORB enrichment peak in ChIP‐seq assay, 174 potential RORB target genes were identified, while 34 of which showed significant correlation with clinical outcomes in a cohort of 498 (GSE62564) NB patients (Figure 2C), including top ranking ones involved in regulation of NF‐κB signaling pathway, such as *NR1D1* and *RIOK3* (Figure 2D,E). By analyzing with dataset from ChIPBase database (https://rnasysu.com/chipbase3), 1410 potential NF‐κB targets were found in RNA‐seq results of SK‐N‐BE(2) cells following stable *RORB* transfection, while 44 of them were lysosome‐related genes, such as *FLCN* and *FNIP1* (Figure 2E). Notably, RORB enrichment peaks were noted at the promoter regions of *NR1D1* and *RIOK3* (Figure 2F). In cultured NB cell lines SK‐N‐BE(2), IMR‐32, SH‐SY5Y, and SK‐N‐AS, endogenous RORB enrichment was validated at promoter regions of *NR1D1* and *RIOK3*, but not of *FLCN* and *FNIP1* (**Figure** **3**A; Figure S4A, Supporting Information). Forced over‐expression or silencing of *RORB* directly increased or decreased its occupancy at the promoter regions of *NR1D1* and *RIOK3* (Figure S4B, Supporting Information), leading to elevation or reduction in both promoter activity and expression levels of *NR1D1* or *RIOK3* (Figure 3B; Figure S4C,D, Supporting Information). In addition, melatonin, a gatekeeper of circadian clocks secreted by pineal gland,^[^ ^12^ ^]^ induced up‐regulation of *RORB* and facilitated the circadian transcript levels of *NR1D1* and *RIOK3*, which was abolished by knockdown of *RORB* (Figure S4E, Supporting Information).

Since previous studies show the repressive roles of *NR1D1* and *RIOK3* in phosphorylation of inhibitor of nuclear factor kappa B kinase alpha/beta (IKKα/β) ^[^ ^13^ ^]^ and inhibitor of nuclear factor kappa B alpha (IκBα),^[^ ^14^ ^]^ respectively, rescue experiments were undertaken to explore the impact of *RORB* and target genes on NF‐κB signaling. Notably, in SK‐N‐BE(2) and IMR‐32 cells stably over‐expressing *RORB*, silencing of *NR1D1* or *RIOK3* prevented the decrease in the phosphorylation of key NF‐κB signaling components IKKα/β (Ser176/180), IκBα (Ser32/36), and p65 (S536) (Figure 3C; Figure S5A–C, Supporting Information). Notably, the enrichment of NF‐κB p65 on promoter regions of *FLCN* and *FNIP1*, but not of *NR1D1* or *RIOK3*, was decreased in these NB cells with stable *RORB* over‐expression, which was reversed by knockdown of *NR1D1* or *RIOK3* (Figure 3D; Figure S5D, Supporting Information). In addition, the decreased NF‐κB activity coupled with up‐regulation of *FLCN* and *FNIP1* in *RORB* over‐expressing NB cells were rescued by silencing of *NR1D1* or *RIOK3* (Figure 3C,D; Figure S5C,D, Supporting Information). Consistent with the established functions of *FLCN* and *FNIP1* as critical regulators of lysosomal biogenesis,^[^ ^15^ ^]^ ectopic expression of *RORB* reduced the levels of lysosome‐associated membrane protein l (LAMP1) and Lyso‐Tracker Red‐positive lysosomes, along with decrease of cathepsin D (CTSD) activity, in SK‐N‐BE(2) and IMR‐32 cells, which was prevented by knockdown of *NR1D1* or *RIOK3* (Figure 3C,E–G; Figure S5C, E–G, Supporting Information). Moreover, ectopic expression of *p65* rescued the alteration of NF‐κB signaling, p65 enrichment and activity, *FLCN* and *FNIP1* expression, lysosomal biogenesis, and CTSD activity in NB cells stably over‐expressing *RORB* (Figure S6A–E, Supporting Information). Pharmacological inhibition of lysosomal function using bafilomycin A1 (BafA1) ^[^ ^16^ ^]^ completely abrogated the increase of proliferative and invasive capacities induced by stable *RORB* knockdown in NB cells (Figure S6F,G, Supporting Information). These data indicated that *RORB* repressed NF‐κB signaling and lysosomal biogenesis via up‐regulation of *NR1D1* and *RIOK3* in NB cells.

### 2.3 RBM10 Directly Interacts with RORB Protein in Liquid Condensates

**Figure 4:**
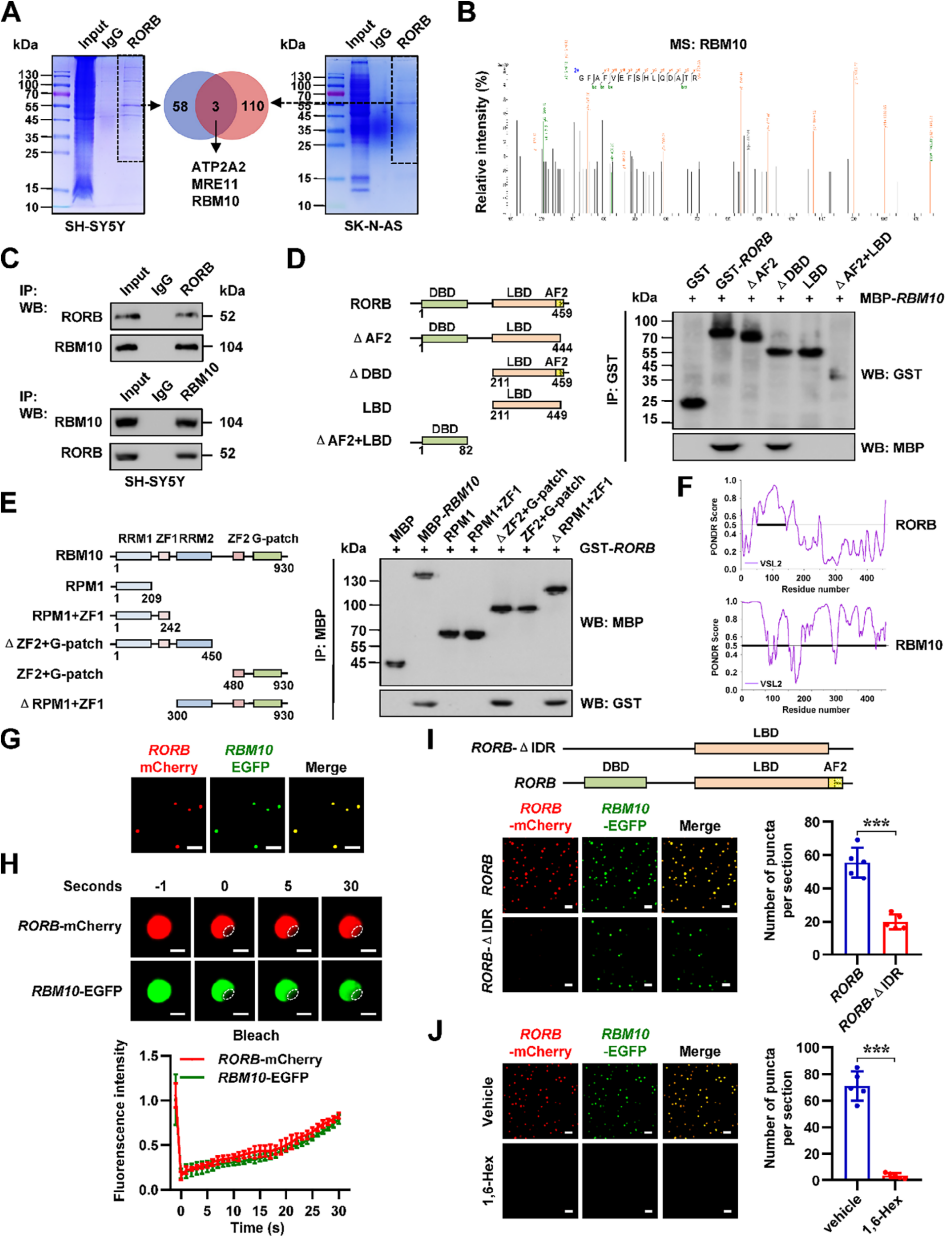
RBM10 directly interacts with RORB protein in liquid condensates. A) Mass spectrometry (MS) assay of indicated electrophoretic bands in Coomassie blue staining (left and right panels) and Venn diagram (middle panel) indicating differential proteins pulled down by RORB specific antibody from lysates of SH‐SY5Y and SK‐N‐AS cells. B) Mass spectrometry assay showing the peptide of RBM10 pulled down by RORB specific antibody from SH‐SY5Y cell lysates. C) Co‐IP and western blot assays revealing endogenous interaction of RORB with RBM10 in SH‐SY5Y cells, with IgG as a negative control. D and E) Illustration (left panel), co‐IP and western blot (right panel) assays indicating the interaction between recombinant full‐length or truncated GST‐tagged RORB and MBP‐tagged RBM10 proteins. F) IDR within RORB and RBM10 proteins analyzed by PONDR (http://www.pondr.com/) program. G) Representative images of droplet formation of recombinant RORB‐mCherry and RBM10‐EGFP proteins in droplet formation buffer. Scale bars: 10 µm. H) Representative images (upper panel) and quantification (lower panel) of FRAP assay showing the exchange kinetics of RORB‐mCherry and RBM10‐EGFP within condensates. Scale bars: 10 µm. I) Representative images (left panel) and quantification (right panel) indicating the droplet formation of wild‐type or IDR‐deficient RORB‐mCherry and RBM10‐EGFP in vitro (*n* = 5). Scale bars: 10 µm. J) Representative images (left panel) and quantification (right panel) showing the liquid droplet formation of RORB‐mCherry and RBM10‐EGFP with incubation of vehicle or 10% 1,6‐hexanediol (1,6‐Hex, *n* = 5). Scale bar, 10 um. Student's *t* test compared the difference in I and J. Data are shown as mean ± s.e.m. (error bars); ***, *p* < 0.001.

To systematically identify RORB‐interacting proteins, we performed co‐immunoprecipitation (co‐IP) coupled with liquid chromatography‐mass spectrometry (LC‐MS/MS) analysis. Proteomic profiling revealed 61 and 113 significantly proteins enriched by RORB specific antibody from SH‐SY5Y and SK‐N‐AS cells, respectively (**Figure** **4**A and Table S2, Supporting Information). Overlapping analysis identified three potential RORB‐binding partners across both cell lines (Figure 4A), including ATPase sarcoplasmic/endoplasmic reticulum Ca^2+^ transporting 2 (ATP2A2), homolog‐double strand break repair nuclease (MRE11), and RNA binding motif protein 10 (RBM10). Among them, only *RBM10* was correlated with reduced survival in a NB cohort of 498 patients (GSE62564). Notably, RT‐PCR using specific primer sets revealed the abundance of *RBM10* variant 1, but not *RBM10* variants 2–5, in NB cells (Figure S7A, Supporting Information). In cultured NB cell lines, *RBM10* expression was up‐regulated (Figure S7B, Supporting Information). Validation studies using co‐IP followed by western blot analysis confirmed the endogenous interaction between RORB and RBM10 in both SH‐SY5Y and SK‐N‐AS cell lines (Figure 4C; Figure S7C, Supporting Information). Domain mapping experiments using purified glutathione S‐transferase (GST)‐tagged RORB and maltose binding protein (MBP)‐tagged RBM10 proteins revealed that activation function 2 (AF2) domain [445–450 amino acids (aa)] of RORB was required for RBM10 binding (Figure 4D; Figure S7D, Supporting Information). Meanwhile, RNA recognition motif 2 (RRM2, 300–384 aa), but not RNA recognition motif 1 (RRM1, 129–209 aa), zinc finger 1 (ZF1, 212–242 aa), zinc finger 2 (ZF2, 759–784 aa), or G patch (858‐904 aa) domain, mediated the binding of RBM10 to RORB (Figure 4E; Figure S7D, Supporting Information), which were further validated by results from SK‐N‐BE(2) cells expressing Flag‐tagged *RORB* and Myc‐tagged *RBM10* (Figure S7E, Supporting Information). Neither forced over‐expression nor genetic knockdown of *RORB* significantly altered *RBM10* expression levels in NB cells, and the effects of *RBM10* on *RORB* levels were similar (Figure S8A, Supporting Information). However, the transactivation of RORB was decreased or increased in NB cells stably over‐expressing or silencing *RBM10* (Figure S8B, Supporting Information). In bimolecular fluorescence complementation (BiFC) assay,^[^ ^17^ ^]^ there was distinct fluorescence of RORB and RBM10 within the nucleus of SK‐N‐BE(2) cells, while mutation in AF2 domain of *RORB* or RRM2 domain of *RBM10* abolished these effects (Figure S8C,D, Supporting Information). Bioinformatic analysis using the PONDR program ^[^ ^18^ ^]^ identified substantial intrinsically disordered regions (IDRs) in both RORB and RBM10 (Figure 4F), prompting us to investigate their capacity for liquid‐liquid phase separation (LLPS). High‐resolution fluorescence microscopy demonstrated that purified recombinant proteins (RORB‐mCherry and RBM10‐EGFP, >90% purity) spontaneously formed biomolecular condensates in vitro (Figure 4G). To characterize the dynamic properties of these condensates, we performed fluorescence recovery after photobleaching (FRAP) assay to demonstrate rapid fluorescence recovery for both RORB‐mCherry and RBM10‐EGFP within the droplets (Figure 4H), indicative of liquid‐like molecular mobility. Deletion of IDR completely abrogated the phase separation capacity of recombinant RORB‐mCherry protein in vitro, and reduced its liquid droplets with RBM10‐EGFP proteins (Figure 4I). Treatment with 1,6‐hexanediol (1,6‐Hex), an established LLPS inhibitor,^[^ ^19^ ^]^ dissociated the liquid droplets of RORB‐mCherry and RBM10‐EGFP proteins (Figure 4J). Collectively, these findings demonstrated that RBM10 physically associated with RORB within liquid biomolecular condensates.

### 2.4 *RBM10* Promotes NB Progression via Repressing *RORB* Transactivation in Liquid Condensates

**Figure 5:**
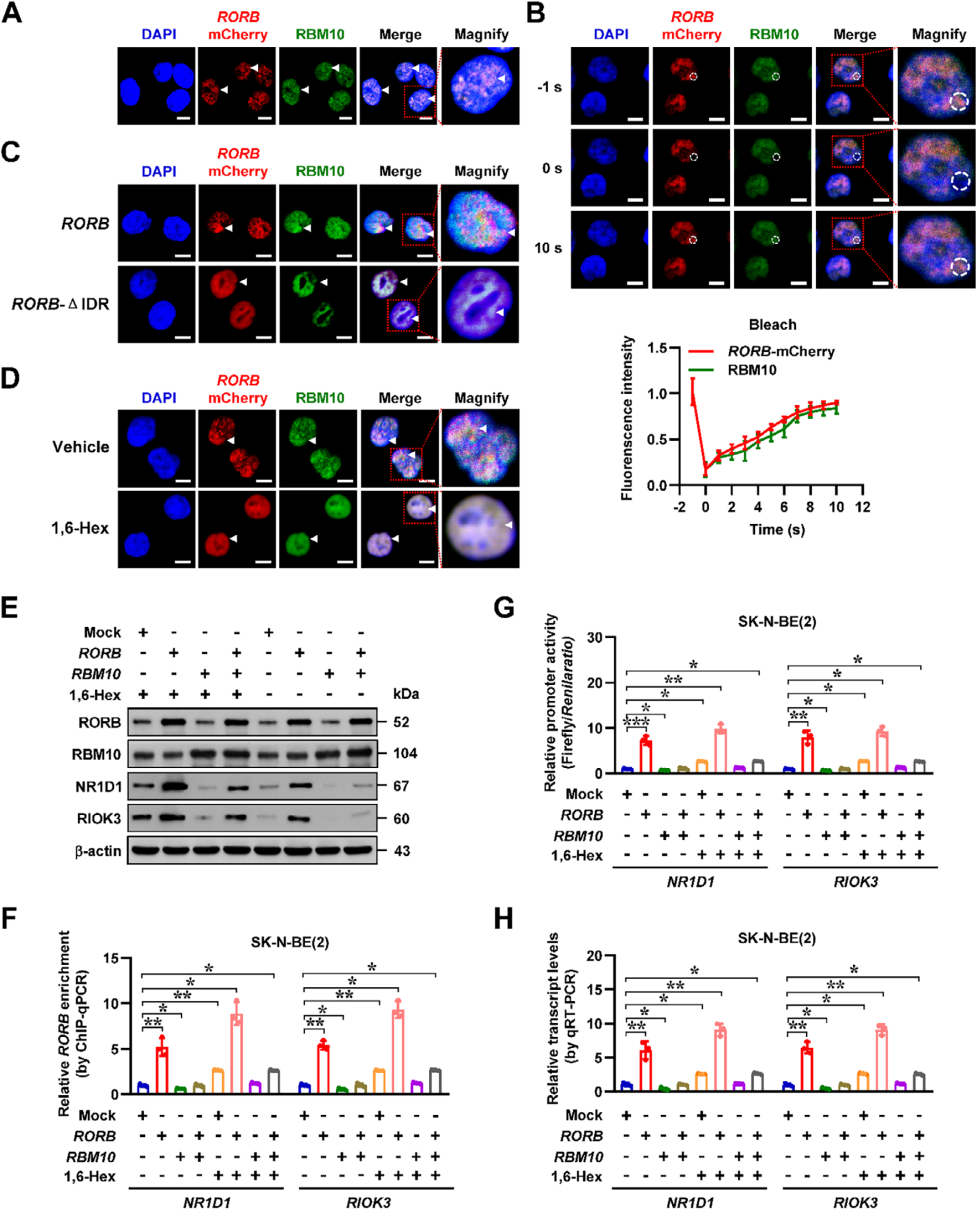
RBM10 represses RORB transactivation in liquid condensates to inhibit downstream gene expression. A) Fluorescence imaging assay indicating the condensate formation (arrowheads) of RORB‐mCherry and RBM10 in SK‐N‐BE(2) cells stably transfected with *RORB* construct. Scale bars: 10 µm. B) Representative images (upper panel) and quantification (lower panel, *n* = 3) of FRAP assay showing the exchange kinetics (arrowheads) of RORB‐mCherry and RBM10 in SK‐N‐BE(2) cells stably transfected with *RORB* construct. Scale bars: 10 µm. C) Fluorescence imaging assay indicating the condensate formation (arrowheads) of RORB‐mCherry and RBM10 in SK‐N‐BE(2) cells stably transfected with full‐length or IDR‐deficient *RORB* construct. Scale bars: 10 µm. D) Representative images of condensate formation (arrowheads) of RORB‐mCherry and RBM10 in SK‐N‐BE(2) cells stably transfected with *RORB* construct, and incubated with vehicle or 10% 1,6‐Hex. Scale bars: 10 µm. E) Western blot assay revealing the expression levels of RORB, RBM10, NR1D1, and RIOK3 in SK‐N‐BE(2) cells stably transfected with empty vector (mock), *RORB*, or *RBM10*, and incubated with vehicle or 10% 1,6‐Hex. F‐H) ChIP‐qPCR (F, normalized to input, *n* = 3), dual‐luciferase (G, *n* = 3), and real‐time qRT‐PCR (H, normalized to *β‐actin*, *n* = 3) assays showing the RORB enrichment, promoter activity, and transcript levels of *NR1D1* and *RIOK3* in SK‐N‐BE(2) cells stably transfected with mock, *RORB*, or *RBM10*, and incubated with vehicle or 10% 1,6‐Hex. One‐way ANOVA compared the difference in F‐H. Data are shown as mean ± s.e.m. (error bars); *, *p* < 0.05; **, *p* < 0.01; ***, *p* < 0.001.

To further investigate the LLPS of RORB‐mCherry and RBM10 proteins in vivo, fluorescence imaging assay was undertaken to reveal their similar compartmentation in SH‐SY5Y cells (**Figure** **5**A), which exhibited fast recovery after photobleaching (Figure 5B). Transfection of RORB‐mCherry construct with deletion of IDR abolished its LLPS in vivo (Figure 5C). Notably, 1,6‐Hex treatment reduced the LLPS of RORB‐mCherry and RBM10 proteins in NB cells (Figure 5D). Over‐expression of *RBM10* neutralized the increase in expression levels of *NR1D1* and *RIOK3* induced by ectopic expression of *RORB* in SK‐N‐BE(2) cells (Figure 5E). Meanwhile, 1,6‐Hex treatment relieved the RBM10‐repressed RORB enrichment (Figure 5F), leading to additional increase in promoter activation, transcriptional levels, and protein expression of *NR1D1* and *RIOK3* in SK‐N‐BE(2) cells (Figure 5E,G,H)

To investigate the functional relationship between RBM10 and RORB in NB cell aggressiveness, we conducted a series of rescue experiments. Ectopic expression of *RBM10* prevented the increase of *FLCN* or *FNIP1* expression, and decrease of LAMP1 levels, lysosome number, and CTSD activity in SK‐N‐BE(2) cells following stable *RORB* over‐expression (Figure S8E–G, Supporting Information). Meanwhile, the decrease in growth and invasive capacity of SK‐N‐BE(2) cells stably over‐expressing *RORB* were restored by ectopic expression of *RBM10* (Figure S8H,I, Supporting Information). In nude mice xenograft models, stable *RBM10* over‐expression effectively counteracted *RORB*‐repressed tumor promotion, reversing its effects on tumor growth, final mass, and Ki‐67 labeling index in SK‐N‐BE(2)‐derived tumors (Figure S9A–C, Supporting Information), accompanied by corresponding alteration in downstream gene expression (Figure S9D, Supporting Information). In addition, nude mice receiving tail vein injections of *RBM10*‐overexpressing SK‐N‐BE(2) cells developed significantly more lung metastatic foci and presented less survival possibility compared to controls, and this enhanced metastatic potential was completely reversed by *RORB* co‐expression (Figure S9E–H, Supporting Information). Above findings indicated that *RBM10* promoted NB progression via repressing *RORB* transactivation in liquid condensates.

### 2.5 Targeting RBM10‐Repressed RORB Transactivation Suppressed the Progression of NB

**Figure 6:**
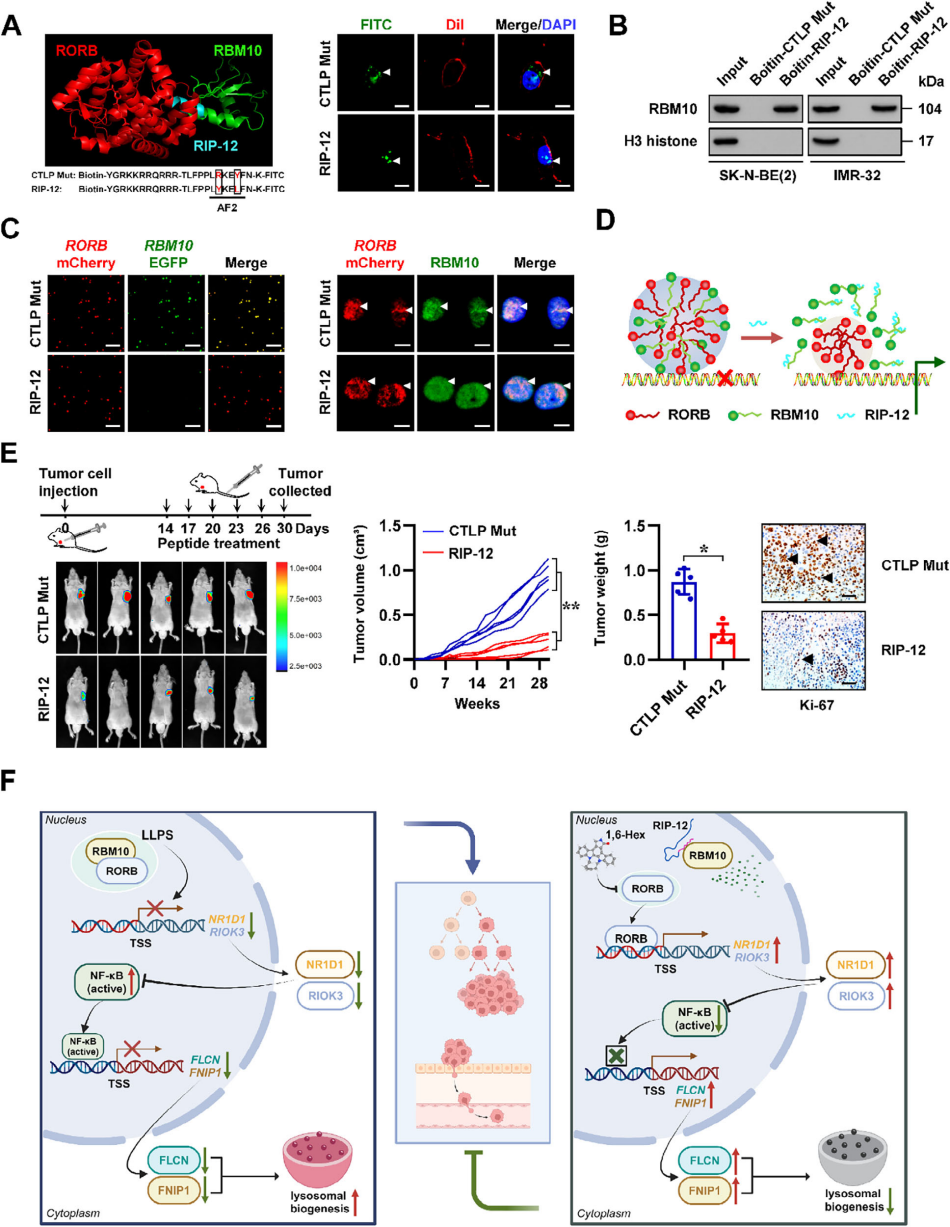
Blocking RBM10‐RORB interaction suppresses the progression of NB. A) Three dimension structure (left panel) showing the inhibitory peptide (RIP‐12) targeting the interaction between RBM10 and RORB protein. Confocal images (right panel) revealing the distribution (arrowheads) of FITC‐labeled control peptide mutant (CTLP Mut) or RIP‐12 (10 µmol L^−1^) within IMR‐32 cells, with nuclei and cellular membranes staining with DAPI or Dil, respectively. Scale bars: 10 µm. B) Peptide pull‐down and western blot assays indicating the interaction of RIP‐12 with RBM10 in SK‐N‐BE(2) and IMR‐32 cell lystaes. C) Representative images of in vitro droplet formation (left panel) of RORB‐mCherry and RBM10‐EGFP, with incubation with CTLP Mut or RIP‐12 (10 µmol L^−1^). Fluorescence imaging assay (right panel) indicating the condensate formation (arrowheads) of RORB‐mCherry and RBM10 in IMR‐32 cells stably transfected with *RORB* construct, and incubated with CTLP Mut or RIP‐12 (10 µmol L^−1^). Scale bars: 10 µm. D) Schematic illustration of the effects of RIP‐12 on RBM10‐RORB interaction and gene transcription. E) Representative images (left panel), growth curve (middle panel), weight (middle panel), and Ki‐67 immunostaining (right panel, arrowheads) of subcutaneous xenograft tumors formed by IMR‐32 cells in nude mice receiving tail vein injection of administration of CTLP Mut or RIP‐12 (3 mg kg^−1^, *n* = 5 per group). F) Schematic depicting the mechanisms underlying *RBM10*/*RORB*‐regulated NF‐κB activity and tumor progression: in liquid condensates, RBM10 interacts with and represses the transactivation of RORB, resulting in down‐regulation of *NR1D1* and *RIOK3*, subsequent activation of NF‐κB signaling, repression of *FLCN* and *FNIP1* expression, and lysosomal biogenesis of NB cells. Meanwhile, blocking the RBM10‐RORB interaction by RIP‐12 is able to suppress lysosomal biogenesis and tumor progression. One‐way ANOVA or Student's *t*‐test compared the difference in E. Data are shown as mean ± s.e.m. (error bars); *, *p* < 0.05; **, *p* < 0.01.

We further illuminated the therapeutic potential targeting interaction between RBM10 and RORB. Based on AF2 domain (445–450 aa) of RORB meditating its interaction with RBM10, a cell‐penetrating peptide was designed and named RBM10 inhibitory peptide with length of 12 amino acids (RIP‐12, **Figure** **6**A). Administration of RIP‐12 or control mutant peptide (CTLP Mut) led to their significant accumulation in nuclei of IMR‐32 cells (Figure 6A). The binding of RIP‐12 to RBM10 was confirmed through a biotinylated peptide pull‐down assay (Figure 6B). Incubation with RIP‐12, but not with CTLP Mut, attenuated the in vitro and in vivo LLPS of RBM10 (Figure 6C), and inhibited the interaction of RBM10 with RORB in SK‐N‐BE(2) or IMR‐32 cells (Figure S10A, Supporting Information), implying its application in modulating RBM10‐repressed RORB activity (Figure 6D). Treatment with RIP‐12 significantly increased or decreased the activity of RORB or NF‐κB (Figure S10B, Supporting Information), respectively, leading to increase in circadian transcript levels of *NR1D1* and *RIOK3*, but not of *RORB* (Figure S10C, Supporting Information). In addition, the protein levels of NR1D1, RIOK3, FLCN, and FNIP1 were up‐regulated by RIP‐12 treatment in IMR‐32 cells (Figure S10D, Supporting Information), along with decrease of LAMP1 levels, lysosome number, and CTSD activity (Figure S10D–G, Supporting Information).

We next investigated whether RIP‐12 influences the aggressiveness of NB cells. Administration of RIP‐12 significantly suppressed NB cell viability in both time‐ and concentration‐dependent manners, while showing no inhibitory effect on HEK‐293 cells (Figure S11A,B, Supporting Information). RIP‐12 treatment markedly reduced both anchorage‐independent growth and invasive capacity of NB cell lines SK‐N‐BE(2) and IMR‐32 (Figure S11C,D, Supporting Information). Administration of RIP‐12 inhibited the growth of IMR‐32 cell‐derived xenograft tumors, as evidenced by reduction in tumor volume, weight, and Ki‐67 proliferative activity (Figure 6E; Figure S11E, Supporting Information), along with up‐regulation of NR1D1, RIOK3, FLCN, or FNIP1 as well as down‐regulation of LAMP1 (Figure S11F, Supporting Information). In addition, RIP‐12 treatment led to increase in body weight of nude mice bearing IMR‐32‐formed xenograft tumors (Figure S11G, Supporting Information), without obvious morphological changes of heart, liver, or kidney tissues (Figure S11H, Supporting Information). Taken together, these data suggested that targeting RBM10‐repressed RORB transactivation was able to suppress progression of NB.

### 2.6 *RBM10*/*RORB* Transcriptional Targets Predict Clinical Outcomes in NB Patients

We further investigate the clinical significance of *RBM10*/*RORB* axis and their target genes. Immunohistochemistry indicated that when compared with ganglion cell neuroblastoma (GNB), lower immunostaining of RORB was concomitant with elevated RBM10 expression in NB tissues (Figure S12A, Supporting Information). Higher levels of *RBM10* was observed in NB specimens, along with down‐regulation of *RORB*, *NR1D1*, *RIOK3*, *FLCN*, or *FNIP1*, especially those with advanced stages (Figure S12B,C, Supporting Information). In a cohort of 498 well‐characterized NB cases (GSE62564), Kaplan‐Meier survival analysis demonstrated that lower expression of *RBM10* (*p* = 2.1 × 10^−7^) and elevated levels of *NR1D1* (*p* = 2.3 × 10^−7^), *RIOK3* (*p* = 1.8 × 10^−6^), *FLCN* (*p* = 7.6 × 10^−20^), or *FNIP1* (*p* = 4.6 × 10^−2^) was correlated with improved clinical outcomes (Figure S12D, Supporting Information). The findings suggested that *RBM10*/*RORB* axis and its downstream targets were linked to NB patients’ outcomes.

## 3 Discussion

Lysosome, the major catabolic organelle with an acidic luminal pH, is able to degrade macromolecule (amino acids or nucleotides) originating from extracellular (endocytosis and phagocytosis) or intracellular (autophagy) sources,^[^ ^20^ ^]^ therefore exerts pivotal functions in diverse cellular processes, including plasma membrane repair, secretion, nutrient (sugars or lipids) signaling, immune response, and ion homeostasis.^[^ ^20^ ^]^ Many types of tumor cells exhibit increased lysosomal abundance and enlarged lysosomal structures compared to normal counterparts, which contribute to drug‐resistance, invasiveness, or metastatic dissemination.^[^ ^21^ ^]^ Lysosome biogenesis depends on the synchronized production of acid hydrolases and their proper delivery through the endocytic transport system. To meet different cellular demands, the expression of lysosomal genes is coregulated by transcription factor EB (TFEB) and transcription factor binding to IGHM enhancer 3 (TFE3), both belonging to the microphthalmia/transcription factor E family of transcription factors.^[^ ^22^ ^]^ Meanwhile, phosphorylation by cyclin‐dependent kinase 4/6 (CDK4/6) triggers cytoplasmic translocation of TFEB and TFE3, effectively suppressing their transcriptional activity.^[^ ^23^ ^]^ Consequently, CDK4/6 inhibition promotes lysosome formation through nuclear accumulation of these factors.^[^ ^23^ ^]^ MYC inhibits lysosome formation through interfering with TFEB/TFE3 recruitment to lysosomal gene regulatory elements.^[^ ^24^ ^]^ In addition, bromodomain containing 4 (BRD4) suppresses lysosome gene expression through directly occupying at their promoter regions.^[^ ^25^ ^]^ Previous studies indicate that loss in GAP activity of FLCN‐FNIP results in the release of TFEB from mechanistic target of rapamycin kinase (mTOR), leading to its constant activation.^[^ ^15^ ^]^ Herein, we discover that RORB is a transcriptional regulator repressing NB progression. *RORB* facilitates the expression of *NR1D1* and *RIOK3* to inhibit NF‐κB activity, resulting in derepression of *FLCN* or *FNIP1* and suppression of NB progression. Staining with LysoTracker red reveals a reduced lysosomal number in *RORB* over‐expressing cells, accompanied by decrease in lysosomal enzyme CTSD activity. Meanwhile, RBM10 physically interacts with and represses RORB transactivation in liquid condensates, and possesses oncogenic properties in facilitating tumor growth and aggressiveness (Figure 6F), indicating the crucial roles of *RBM10*/*RORB*/*NF‐κB* axis in NB progression.

*RORB*, consisting of 10 exons and locating at chromosome 9q21.13, was initially characterized as a brain‐specific transcription factor, with high expression levels in the adult retina, suprachiasmatic nucleus, or pineal gland, without abundance in non‐neuronal tissues.^[^ ^26^ ^]^ *RORB* is essential in controlling both proliferation of retinal progenitor cells.^[^ ^27^ ^]^ and differentiation of rod photoreceptor,^[^ ^28^ ^]^ while *RORB^−/−^* mice exhibit blindness or behavioral changes.^[^ ^29^ ^]^ In colorectal cancer, *RORB* is down‐regulated and influences Wnt activities to modulate self‐renewal of colorectal cancer‐initiating cells.^[^ ^8^ ^]^ Yet, the functional significance and molecular mechanisms of *RORB* in other tumor types remain poorly understood. In current study, we found the down‐regulation of *RORB* in NB tissues, including those with diploidy. Previous studies indicate that diploidy is associated with early treatment failure of NB patients, whereas individuals with hyperdiploidy (predominantly near‐triploid) exhibit long‐term survival.^[^ ^30^ ^]^ In diploid and tetraploid NB specimens, higher frequencies of structural chromosome aberrations and *MYCN* amplification are noted than those of near‐triploid tumors.^[^ ^31^ ^]^ However, due to limited case number, the association of *RORB* expression with NB cell ploidy warrants further investigation. Our results illuminate a functional relationship between RORB and NF‐κB activities in NB. Actually, our results demonstrate that RORB, functioning as a transcription factor, directly activates *NR1D1* and *RIOK3* expression in NB cells. *NR1D1*, also known as reverse orientation c‐erbA gene alpha (*REV‐ERBα*), is an established core component of the circadian clock machinery that transcriptionally regulates the levels of key clock genes *BMAL1* and *CLOCK*.^[^ ^32^ ^]^ Beyond its core circadian function, *NR1D1* plays pleiotropic roles in diverse physiological processes including metabolic regulation, immune cell differentiation, and cellular fate determination.^[^ ^7^ ^]^ In breast cancer, NR1D1 binds with poly (ADP‐ribose) polymerase 1 (PARP1) to prevent the assembly of DNA damage response complexes at lesion sites,^[^ ^33^ ^]^ and high *NR1D1* expression correlates significantly with improved overall survival of patients.^[^ ^34^ ^]^ Meanwhile, *RIOK3* belongs to the atypical right open reading frame (RIO) kinase family that promotes actin cytoskeletal organization, while depletion of *RIOK3* inhibits the invasion and metastatic potential of breast or pancreas cancer cells.^[^ ^35^ ^]^ *RIOK3* is highly expressed in glioma and pancreatic cancer,^[^ ^36^ ^]^ while silencing of *RIOK3* inhibits the proliferation, migration, invasion of tumor cells.^[^ ^36a^ ^]^ However, our evidence indicated that down‐regulation of *RIOK3* in NB tissues correlated with poor outcomes of patients, suggesting its functions in a context‐dependent manner. Consistent with prior studies demonstrating that NR1D1 and RIOK3 inhibit IKKα/β phosphorylation.^[^ ^13^ ^]^ or IκBα degradation,^[^ ^14^ ^]^ our studies showed that RORB suppressed NF‐κB signaling activation, at least in part, through up‐regulation of *NR1D1* and *RIOK3*, resulting in reduction of lysosomal biogenesis during NB progression. Meanwhile, the roles of *RORB* in regulating circadian clock‐dependent expression of downstream genes warrant further investigation.

RBM10, a member of RNA binding protein (RBP) family, participates in alternatively splicing and mRNA stabilization,^[^ ^37^ ^]^ and is vital for embryonic development of heart, lungs, kidneys, limbs, and central nervous system.^[^ ^38^ ^]^ Recent evidence indicates tumor suppressive or oncogenic roles of *RBM10* in tumorigenesis.^[^ ^39^ ^]^ Mutation or depletion of *RBM10* is documented in lung, pancreatic, and colorectal cancers.^[^ ^40^ ^]^ Over‐expression of *RBM10* inhibits the viability, colony formation, and cell cycle process of lung cancer cells.^[^ ^41^ ^]^ In contrast, high *RBM10* expression is positively correlated with aggressiveness of metastatic melanoma,^[^ ^42^ ^]^ and associated with increase of vascular endothelial growth factor levels in breast cancer.^[^ ^43^ ^]^ Knockdown of *RBM10* leads to reduction in epithelial to mesenchymal transition via association with filamin A‐binding RhoGTPase‐activating protein (FILGAP), a regulator of cell migration,^[^ ^44^ ^]^ and augments proapoptotic effects of staurosporine in NB cells.^[^ ^45^ ^]^ Nevertheless, the exact mechanisms by which *RBM10* operates in NB, along with its specific functions, are still poorly defined. In this investigation, we employed co‐IP coupled with mass spectrometry to identify RBM10 as a protein partner of RORB. Human RBM10 have two RRM domains, an octamer repeat of aromatic residues (OCRE) domain, a G‐patch domain, and two zinc finger domains.^[^ ^37^ ^]^ Our findings demonstrated that RRM2 domain of RBM10 was essential for its binding to the AF2 domain of RORB, leading to the suppression of RORB transactivation in NB cells. Of importance, *RBM10* was up‐regulated in NB tissues and cells, and exerted oncogenic roles in lysosomal biogenesis and progression of NB via depressing *RORB* transactivation. Blocking RBM10‐RORB interaction was able to suppress malignant behaviors of NB cells, highlighting the importance of *RBM10* and *RORB* in NB progression.

In conclusion, we demonstrate, for the first time, that *RORB* is associated with favorable outcome and promotes regression of NB. Meanwhile, RBM10 suppresses RORB transactivation to promote aggressiveness of NB cells. Mechanistically, as a co‐factor, RBM10 represses the transcriptional activity of RORB in liquid condensates, resulting in down‐regulation of *NR1D1* and *RIOK3* that further activates NF‐κB activity to facilitate lysosomal biogenesis via repressing expression of *FLCN* and *FNIP1*. A peptide blocking interaction between RORB and RBM10 is able to suppress in vitro and in vivo aggressive features of NB cells. Collectively, our findings significantly advance the understanding of lysosomal biogenesis and NB regression mediated by transcription factor networks. Specifically, we demonstrate that *RBM10*/*RORB*/*NF‐κB* axis regulates these processes, highlighting its potential as a novel therapeutic target for tumors. Due to limited case number, the association of *RORB* expression with tumor cell ploidy and prognostic values of *RBM10*/*RORB* axis in NB warrant further investigation via a larger series of cases with longer follow‐up duration. Efforts will be deserved to reveal additional functions (eg. apoptosis induction) of *RORB* during the progression of NB and other tumors. Meanwhile, therapeutic potential and toxicity of RIP‐12 or melatonin in regulating *RBM10*/*RORB* axis deserve studies by using patient‐derived tumor xenografts or immune‐competent mice models.

## 4 Experimental Section

### Cell Lines and Culture

Human cell lines were obtained from American Type Culture Collection (ATCC, Rockville, MD), including HEK‐293 (CRL‐1573), HEK‐293T (CRL‐3216), SH‐SY5Y (CRL‐2266), SK‐N‐AS (CRL‐2137), SK‐N‐SH (HTB‐11), SK‐N‐BE(2) (CRL‐2271), BE(2)‐C (CRL‐2268), IMR‐32 (CCL‐127), and SK‐N‐DZ (CRL‐2149). All cell lines were authenticated through short tandem repeat (STR) profiling and utilized within six months of resuscitation. Routine mycoplasma screening was performed using the MycoAlert PLUS Mycoplasma Detection Kit (Takara, Japan), with all cultures testing negative for contamination. Cell lines SK‐N‐DZ, IMR‐32, SK‐N‐AS, SK‐N‐SH, HEK293, and HEK‐293T were maintained at 37 °C under 5% CO_2_ in a humid environment using Dulbecco's Modified Eagle's Medium (DMEM; Invitrogen, Carlsbad, CA). Meanwhile, SK‐N‐BE(2), BE(2)‐C, and SH‐SY5Y cells were cultured under identical temperature and CO_2_ conditions, utilizing either Minimum Essential Medium (MEM)/F12 or DMEM/F12 (Invitrogen) supplemented with 10% fetal bovine serum (FBS; Sigma, St. Louis, MO). To achieve synchronization in vitro, cells were incubated with dexamethasone (100 nmol L^−1^, Sigma, St. Louis, MO) for 60 min, and subsequently maintained in complete growth medium.^[^ ^46^ ^]^ Twenty‐four hours after synchronization, cells were harvested at seven sequential time points (4 h intervals).

### Western Blotting

Proteins were prepared from tissues or cultured cells using 1× RIPA lysis buffer (Promega, Madison, WI). Western blot analysis was conducted following standard protocols,^[^ ^46^, ^47^ ^]^ using antibodies specific against RORB (ab228650), RBM10 (ab72423), NR1D1 (ab174309), RIOK3 (ab241361), phospho‐IKKα/β (p‐IKKα/β at Ser176/180, ab17943), IKKα (ab32041), IKKβ (ab124957), phospho‐IκBα (p‐IκBα at Ser32/36, ab133462), IκBα (ab32518), phospho‐p65 (p‐p65 at Ser536, ab76302), p65 (ab32536), FLCN (ab124885), FNIP1 (ab215725), LAMP1 (ab278043), Flag‐tag (ab125243), Myc‐tag (ab206486), GST (ab19256), MBP (ab119994), histone H3 (ab5103), or β‐actin (ab7291, Abcam Inc., Cambridge, MA). The specificity of commercial antibodies was validated by gene over‐expression or silencing experiments, with isotype IgG as a negative control.

### Chromatin Immunoprecipitation (ChIP) and ChIP‐Seq

ChIP assay was performed with the EZ‐ChIP kit (Millipore, Burlington, MA),^[^ ^46^, ^47^ ^]^ and antibodies against RORB (Abcam Inc., ab228650) or NF‐κB p65 (ab218533). Two confluent 55 cm^2^ dishes (1 × 10^7^ cells per dish) were collected, while formaldehyde was added dropwise to the media (0.75% final concentration) for 10 min to cross‐link proteins to DNA, with incubation under gentle rotation. Following treatment with glycine (125 mmol L^−1^ final concentration) and two washes with 10 ml of ice‐cold phosphate‐buffered saline (PBS), cells were centrifuged to form pellets and then reconstituted in ChIP lysis buffer (Thermo Fisher Scientific, Inc., Waltham, MA; 750 µl per 1 × 10⁷ cells). Chromatin fragmentation to 200–500 bp was achieved by sonicating lysates on ice using 20 s pulses. Sheared chromatin samples were then subjected to immunoprecipitation through overnight incubation with 3 µg of target‐specific antibodies or control IgG. Captured immuno‐complexes underwent sequential rinsing with ChIP wash solution (Thermo Fisher Scientific, Inc.) followed by elution in a buffer containing 1.0% sodium dodecyl sulfate (SDS) and 1.0 mol L^−1^ NaHCO_3_. Real‐time quantitative PCR (qPCR) was utilized with SYBR Green PCR Master Mix (Takara) along with primers (Table S3, Supporting Information). Isotype IgG was used as a negative control to standardize the immunoprecipitated DNA. For ChIP‐seq, after library preparation, Illumina HiSeq X Ten was used for transcriptome sequencing (Wuhan SeqHealth Technology Co., Ltd., China). The 100‐bp paired‐end sequences were aligned to genomic features with HTSeq v0.6.0, and the number of transcript fragments (FPKM) per million mapped fragments was analyzed. The dataset was submitted to Gene Expression Omnibus (GEO), under accession GSE305134.

### RNA Sequencing (RNA‐seq)

Total RNA was extracted from 1 × 10^6^ cells with RNeasy Mini Kit (Qiagen). RNA‐seq libraries were prepared by Wuhan SeqHealth Technology Co., Ltd. following standard Illumina protocols, with subsequent sequencing on the HiSeq X Ten platform to generate 100 nucleotide paired‐end reads. Gene‐level read counts were quantified using HTSeq (version 0.6.0) with default union‐counting mode, followed by FPKM normalization to account for transcript length and sequencing depth variations. All sequencing datasets were accessible through GEO database (GSE305133).

### Co‐IP and Mass Spectrometry

Co‐IP assay was carried out by previously published methodology,^[^ ^46^, ^47^ ^]^ with antibodies specific for RORB (ab228650), RBM10 (ab72423), Flag‐tag (ab125243), or Myc‐tag (ab206486, Abcam Inc., Cambridge, MA). Bead‐bound proteins were released, separated using SDS‐polyacrylamide gel electrophoresis (PAGE), and detected via Coomassie blue staining, immunoblotting, or proteomic profiling (Wuhan SpecAlly Life Technology Co., Ltd, China).

### Cellular Viability, Growth, and Invasion Assays

Cellular viability, growth, and invasion in vitro was measured via the colorimetric assay with tetrazolium bromide (MTT, MerkMillipore),^[^ ^48^ ^]^ soft agar,^[^ ^46^, ^47^ ^]^ and matrigel invasion.^[^ ^46^, ^47^ ^]^ assays, respectively.

### Immunohistochemical Staining

Immunohistochemical staining was carried out using standard protocols.^[^ ^46^, ^47^ ^]^ and following antibodies: anti‐Ki‐67 (ab92742; 1:100), anti‐RORB (ab188756, Abcam; 1:200), and anti‐RBM10 (ab220847, Abcam; 1:200). Specificity was verified using antigen‐blocking peptide or IgG isotype control. The intensity of staining reaction was evaluated by two pathologists blind to group assignment.

### Statistical Analysis

The data were analyzed by GraphPad 8.0 software (GraphPad Software, Boston, MA). All quantitative findings were expressed as mean ± standard error of the mean (SEM). Gene expression cutoff values were established based on the median expression levels. To quantify the significant concordance of independent gene sets, Fisher's exact test was implemented. To evaluate differences among tumor cells or tissues, statistical comparisons were performed using either non‐parametric Mann‐Whitney U test (for non‐normal distributions), one‐way analysis of variance (ANOVA, for multiple distributed parametric data), or Student's *t*‐test (for normally distributed parametric data). Kaplan‐Meier curves were applied for analyzing survival data, while group comparisons were performed using two‐sided log‐rank test. Statistical significance was assessed by using two‐tailed tests, with significance defined as false discovery rate (FDR)‐adjusted *p*‐value less than 0.05.

## Conflict of Interest

The authors declare no conflict of interest.

## Author Contributions

Y.G., X.W., and C.Y. contributed equally to this work. Y.G. and X.W. conceived and designed the research. Y.G., X.W., C.Y., Z.W., X.W., X.L., J.Q., and S.Z. performed the experiments. Y.G. and C.Y. analyzed the data. Y.G., Q.T. and L.Z. wrote the manuscript. All authors read and approved the final manuscript.

## Supporting information



Supporting Information

## Data Availability

The data that support the findings of this study are available in the supplementary material of this article.
