# Supplementary material for: Targeting RBM10‐Repressed RORB Activity in Liquid Condensates Inhibits Lysosomal Biogenesis and Neuroblastoma Progression via Affecting NF‐κB Signaling
Source: Adv Sci (Weinh). 2025 Sep 3;12(44):e06131. doi: 10.1002/advs.202506131 (PMC12667445; doi:10.1002/advs.202506131)
Supplement: Supplementary file 1 — Supporting Information [file ADVS-12-e06131-s001.pdf]

Supporting Information

Targeting RBM10-Repressed RORB Activity in Liquid Condensates Inhibits Lysosomal Biogenesis and Neuroblastoma Progression via Affecting NF-κB Signaling

Yanhua Guo, *Xiaojing Wang*, *Chunhui Yang*, *Zhijie Wang*, *Xiaolin Wang*, *Xinyue Li*, *Jiaying Qu*, *Shunchen Zhou*, *Liduan Zheng *, Qiangsong Tong **

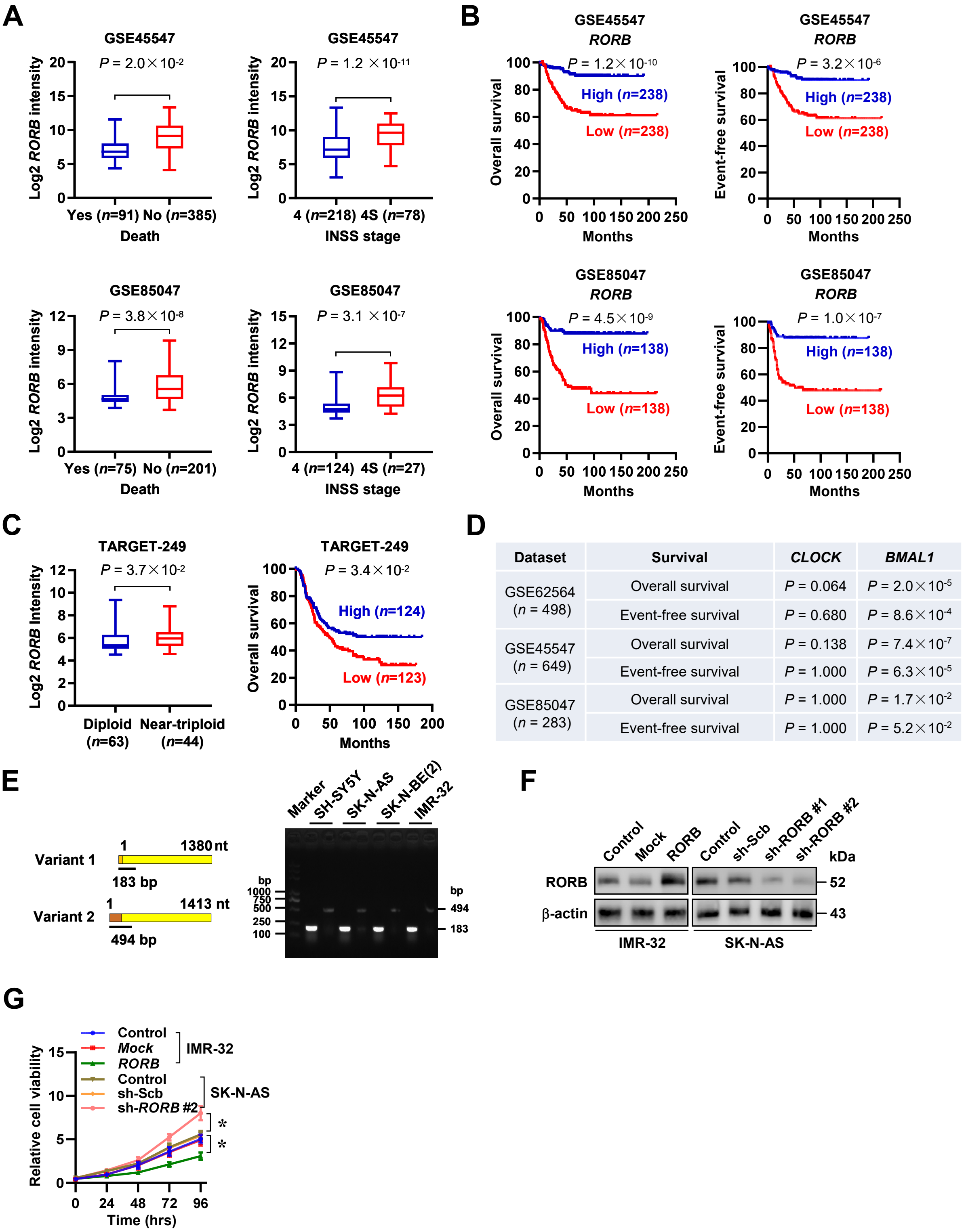

Dataset	Survival	*CLOCK*	*BMAL1*
GSE62564 (*n* = 498)	Overall survival	*P* = 0.064	*P* = 2.0×10^{-5}
	Event-free survival	*P* = 0.680	*P* = 8.6×10^{-4}
GSE45547 (*n* = 649)	Overall survival	*P* = 0.138	*P* = 7.4×10^{-7}
	Event-free survival	*P* = 1.000	*P* = 6.3×10^{-5}
GSE85047 (*n* = 283)	Overall survival	*P* = 1.000	*P* = 1.7×10^{-2}
	Event-free survival	*P* = 1.000	*P* = 5.2×10^{-2}

Figure S1. Tumor suppressive roles of *RORB* in NB. A) Mining of two public datasets revealing the *RORB* levels in 649 (GSE45547) and 283 (GSE85047) NB tissues with different status of death or INSS stages. **B**) Kaplan-Meier curve showing overall or event-free survival of 649 (GSE45547) and 283 (GSE85047) NB patients with high or low *RORB* expression (cutoff values=8.59 and 5.12). **C**) Mining of a public dataset derived from TARGET database (https://ocg.cancer.gov/programs/target/projects/neuroblastoma) showing the *RORB* levels (left panel) in NB tissues with diploid (*n*=63) or near-triploid (*n*=44) status. Kaplan-Meier curve (right panel) indicating overall survival of 249 NB patients with high or low *RORB* expression (cutoff values= 5.33). **D**) Overall or event-free survival of 498 (GSE62564), 649 (GSE45547), and 283 (GSE85047) NB cases with low or high levels of *CLOCK* and *BMAL1*. **E**) RT-PCR assay with specific primer sets revealing the existence of *RORB* variants in cultured NB cell lines. **F**) Western blot assay indicating the expression of RORB in untreated (control) IMR-32 and SK-N-AS parental cells, and those stably transfected with empty vector (mock), *RORB*, scramble shRNA (sh-Scb), sh-*RORB* #1, or sh-*RORB* #2. **G**) MTT colorimetric assay indicating the viability of untreated (control) IMR-32 and SK-N-AS parental cells, and those stably transfected with mock, *RORB*, sh-Scb, or sh-*RORB* #2 (*n*=4). Non-parametric Mann-Whitney U test compared the difference in **A** and **C**. Log-rank test for survival comparison in **B**-**D**. One-way ANOVA compared the difference in **G**. Data are shown as mean ± s.e.m. (error bars); *, $P<0.05$.

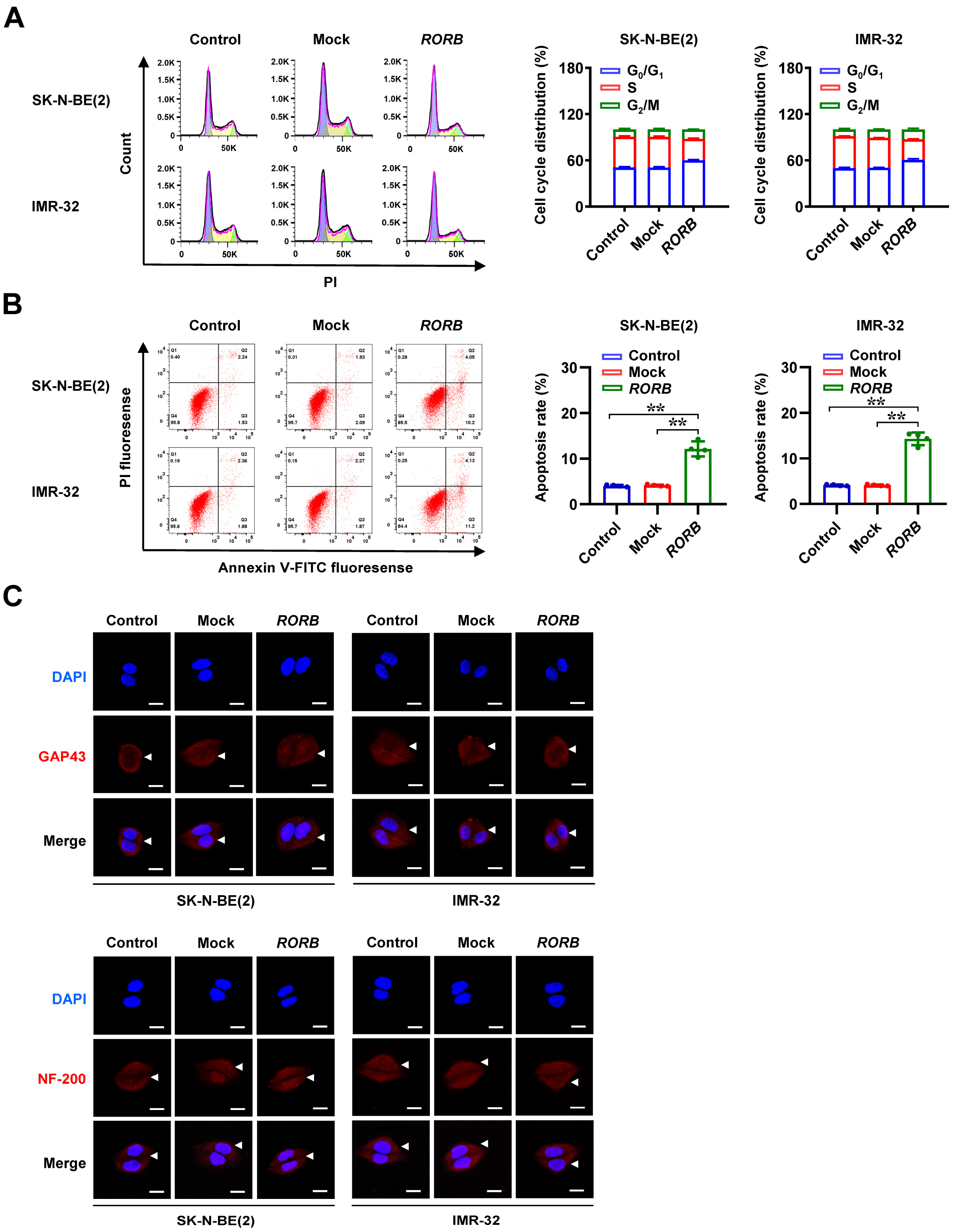

Figure S2. Effects of *RORB* on cell cycle process, apoptosis, and differentiation of NB cells. A) Representative images (left panel) and quantification (right panel) of PI-staining flow cytometry showing the cell cycle phases of untreated (control) SK-N-BE(2) and IMR-32 parental cells, and those stably transfected with empty vector (mock) or *RORB* (n=5). **B**) Representative images (left panel) and quantification (right panel) of Annexin V/PI-staining flow cytometry showing the apoptosis of untreated (control) SK-N-BE(2) and IMR-32 parental cells, and those stably transfected with mock or *RORB* (n=4). **C**) Immunofluorescent assay showing the expression (arrowheads) of neuronal differentiation markers (GAP43 and NF-200) in untreated (control) SK-N-BE(2) and IMR-32 parental cells, and those stably transfected with mock or *RORB*. Scale bars: 10 μm. One-way ANOVA compared the difference in **B**. Data are shown as mean ± s.e.m. (error bars); **, P<0.01.

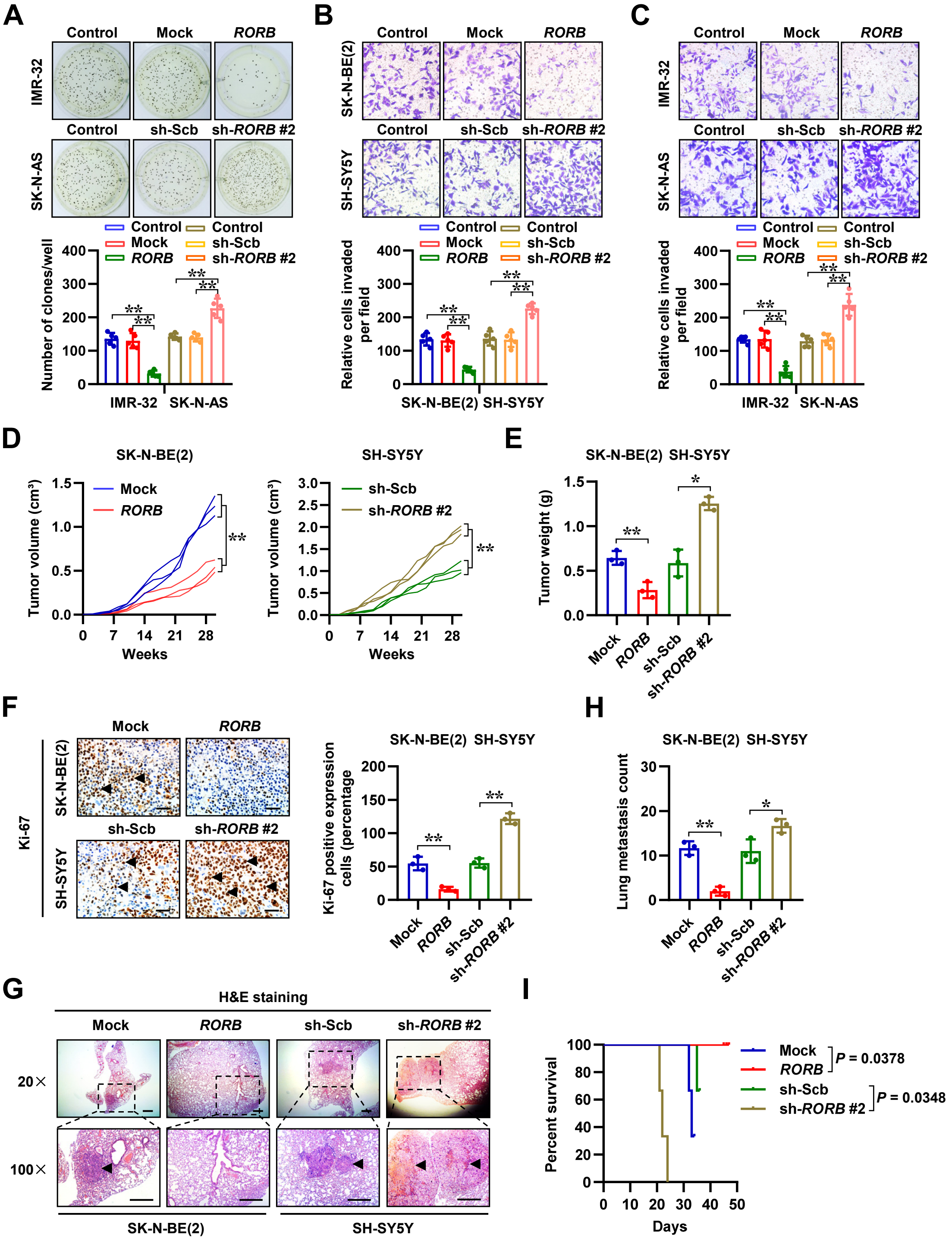

Figure S3. ***RORB* suppresses the growth and metastasis of NB cells *in vitro* and *in vivo*.** **A**) Representative images (upper panel) and quantification (lower panel) of soft agar assay showing the growth of untreated (control) IMR-32 and SK-N-AS parental cells, and those stably transfected with empty vector (mock), *RORB*, scramble shRNA (sh-Scb), or sh-*RORB* #2 (*n*=5). **B** and **C**) Representative images (upper panel) and quantification (lower panel) of matrigel invasion assay showing the invasion of untreated (control) SK-N-BE(2), SH-SY5Y, IMR-32 and SK-N-AS parental cells, and those stably transfected with mock, *RORB*, sh-Scb, or sh-*RORB* #2 (*n*=5). **D** and **E**) *In vivo* growth curve and weight of subcutaneous xenograft tumors in nude mice formed by SK-N-BE(2) or SH-SY5Y cells stably transfected with mock, *RORB*, sh-Scb, or sh-*RORB* #2 (*n*=3 per group). **F**) Representative images (left panel) and quantification (right panel) of Ki-67 immunostaining (arrowheads) in subcutaneous xenograft tumors in nude mice formed by SK-N-BE(2) or SH-SY5Y cells stably transfected with mock, *RORB*, sh-Scb, or sh-*RORB* #2 (*n*=3 per group). **G** and **H**) HE staining (G, arrowheads) and quantification (H) of lung metastasis in nude mice treated with tail vein injection of SK-N-BE(2) or SH-SY5Y cells stably transfected with mock, *RORB*, sh-Scb, or sh-*RORB* #2 (*n*=3 per group). Scale bar, 100 μm. **I**) Kaplan-Meier curves of nude mice treated with tail vein injection of SK-N-BE(2) or SH-SY5Y cells stably transfected with mock, *RORB*, sh-Scb, or sh-*RORB* #2 (*n*=3 per group). One-way ANOVA compared the difference in **A-F** and **H**. Log-rank test for survival comparison in **I**. Data are shown as mean ± s.e.m. (error bars); *, P<0.05; **, P<0.01.

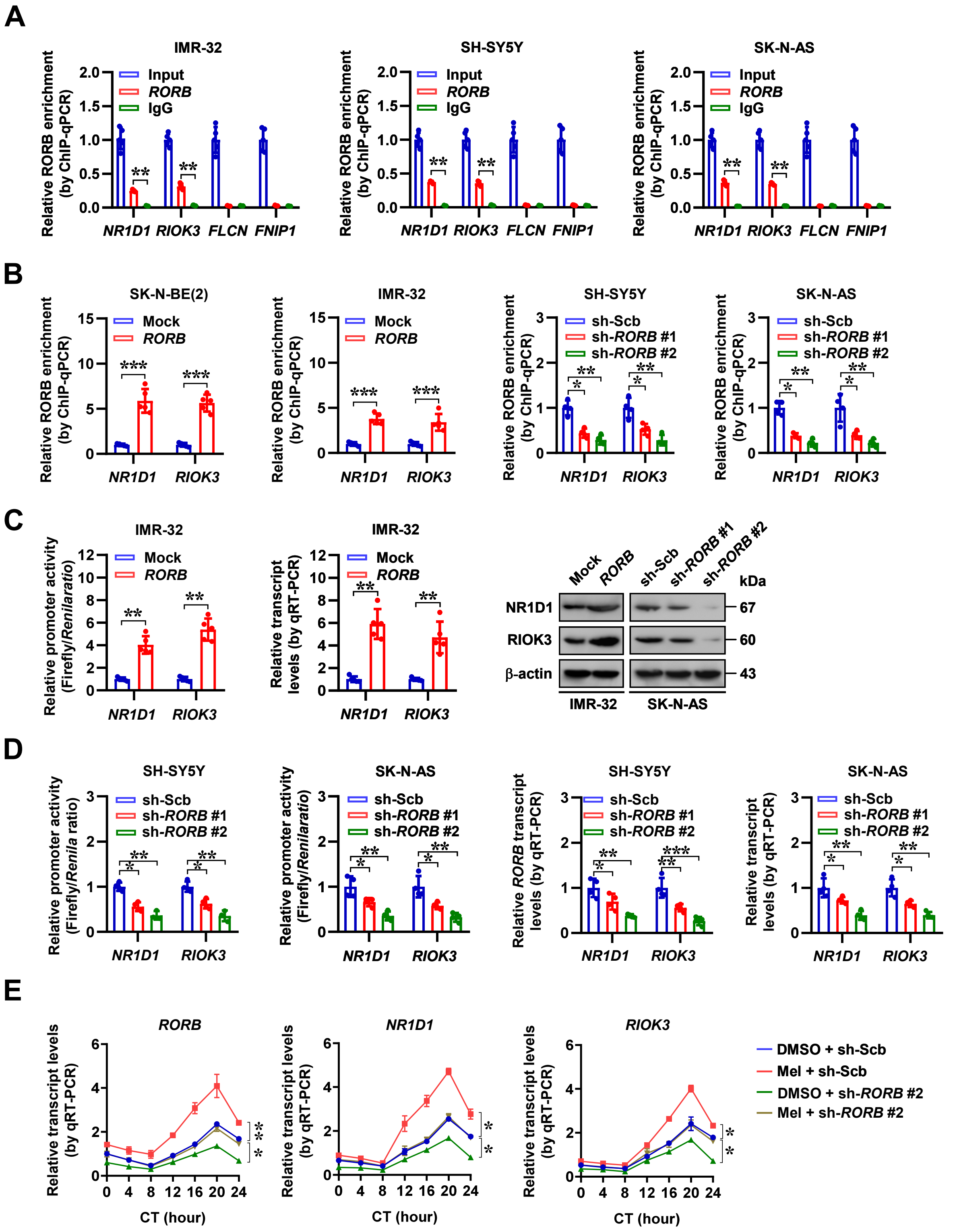

Figure S4. RORB facilitates the expression of *NR1D1* and *RIOK3* in NB cells. A) ChIP-qPCR assay (normalized to input) showing the endogenous enrichment of RORB on promoter regions of *NR1D1*, *RIOK3*, *FLCN*, or *FNIP1* in IMR-32, SH-SY5Y, and SK-N-AS cells (n=5). **B**) ChIP-qPCR assay (normalized to input) revealing the enrichment of RORB on promoter regions of *NR1D1* or *RIOK3* in NB cells stably transfected with empty vector (mock), *RORB*, scramble shRNA (sh-Scb), sh-*RORB* #1, or sh-*RORB* #2 (n=5). **C**) Dual-luciferase (n=5), real-time qRT-PCR (normalized to *β-actin*, n=5), and western blot assays indicating the promoter activity, transcript, and protein levels of *NR1D1* and *RIOK3* in IMR-32 or SK-N-AS cells stably transfected with mock, *RORB*, sh-Scb, sh-*RORB* #1, or sh-*RORB* #2. **D**) Dual-luciferase (n=5) and real-time qRT-PCR (normalized to *β-actin*, n=5) assays showing the promoter activity and transcript levels of *NR1D1* and *RIOK3* in SH-SY5Y and SK-N-AS cells stably transfected with sh-Scb, sh-*RORB* #1, or sh-*RORB* #2. **E**) Real-time qRT-PCR (normalized to *β-actin*, n=5) showing the circadian transcript levels of *RORB*, *NR1D1* and *RIOK3* in SK-N-BE(2) cells treated with melatonin (Mel, 10 nmol$\cdot$L^{-1}), and those stably transfected with sh-Scb or sh-*RORB* #2. Student's t-test or one-way ANOVA compared the difference in **A-E**. Data are shown as mean ± s.e.m. (error bars); *, P<0.05; **, P<0.01; ***, P<0.001.

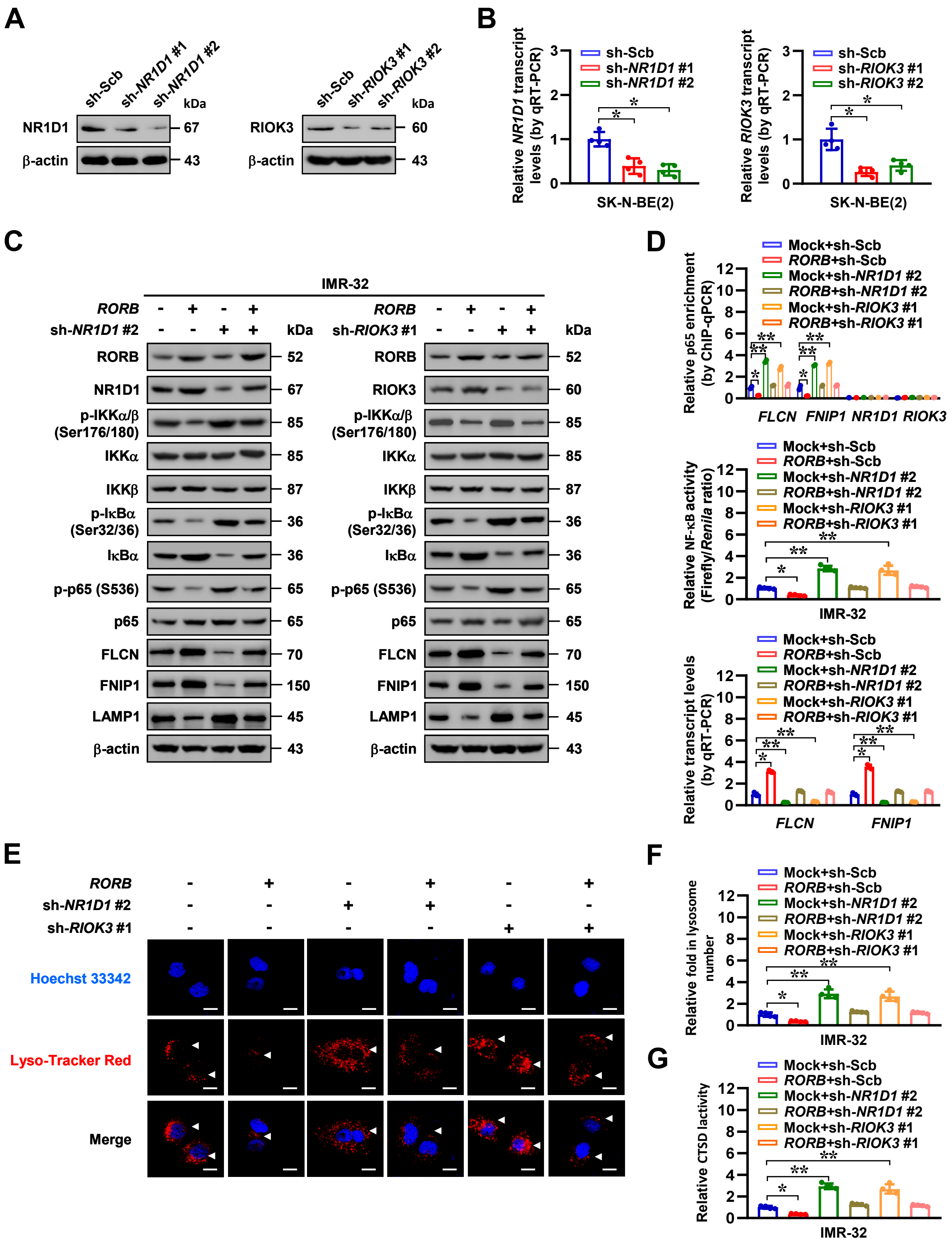

Figure S5. ***RORB* represses the NF-κB signaling and lysosome biogenesis via up-regulation of *NR1D1* and *RIOK3* in NB cells. A** and **B**) Western blot (A) and real-time qRT-PCR (B, normalized to *β-actin*, *n*=5) indicating the expression levels of *NR1D1* and *RIOK3* in SK-N-BE(2) stably transfected with scramble shRNA (sh-Scb), sh-*NR1D1* #1, sh-*NR1D1* #2, sh-*RIOK3* #1, or sh-*RIOK3* #2. **C**) Western blot assay revealing the levels of RORB, NR1D1, RIOK3, phosphorated or non-phosphorated IKKα/β, IκBα, and p65, FLCN, FNIP1, and LAMP1 in IMR-32 cells stably transfected with mock or *RORB*, and those co-transfected with sh-Scb, sh-*NR1D1* #2, or sh-*RIOK3* #1. **D**) ChIP-qPCR (normalized to input, *n*=3), dual-luciferase (*n*=4), and real-time qRT-PCR (normalized to *β-actin*, *n*=4) assays indicating the enrichment or activity of p65 and transcript levels of *FLCN* or *FNIP1* in IMR-32 cells stably transfected with mock or *RORB*, and those co-transfected with sh-Scb, sh-*NR1D1* #2, sh-*RIOK3* #1 (*n*=4). **E** and **F**) Representive images (E) and quantification (F) of fluroscence assay showing Lyso-Tracker Red-positive lysosomes (arrowheads) within IMR-32 cells stably transfected with mock or *RORB*, and those co-transfected with sh-Scb, sh-*NR1D1* #2, or sh-*RIOK3* #1 (*n*=4). Scale bars: 10 μm. **G)** The CTSD activity in IMR-32 cells stably transfected with mock or *RORB*, and those co-transfected with sh-Scb, sh-*NR1D1* #2, or sh-*RIOK3* #1 (*n*=4). One-way ANOVA compared the difference in **B**, **D**, **F** and **G**. Data are shown as mean ± s.e.m. (error bars); *, $P<0.05$; **, $P<0.01$.

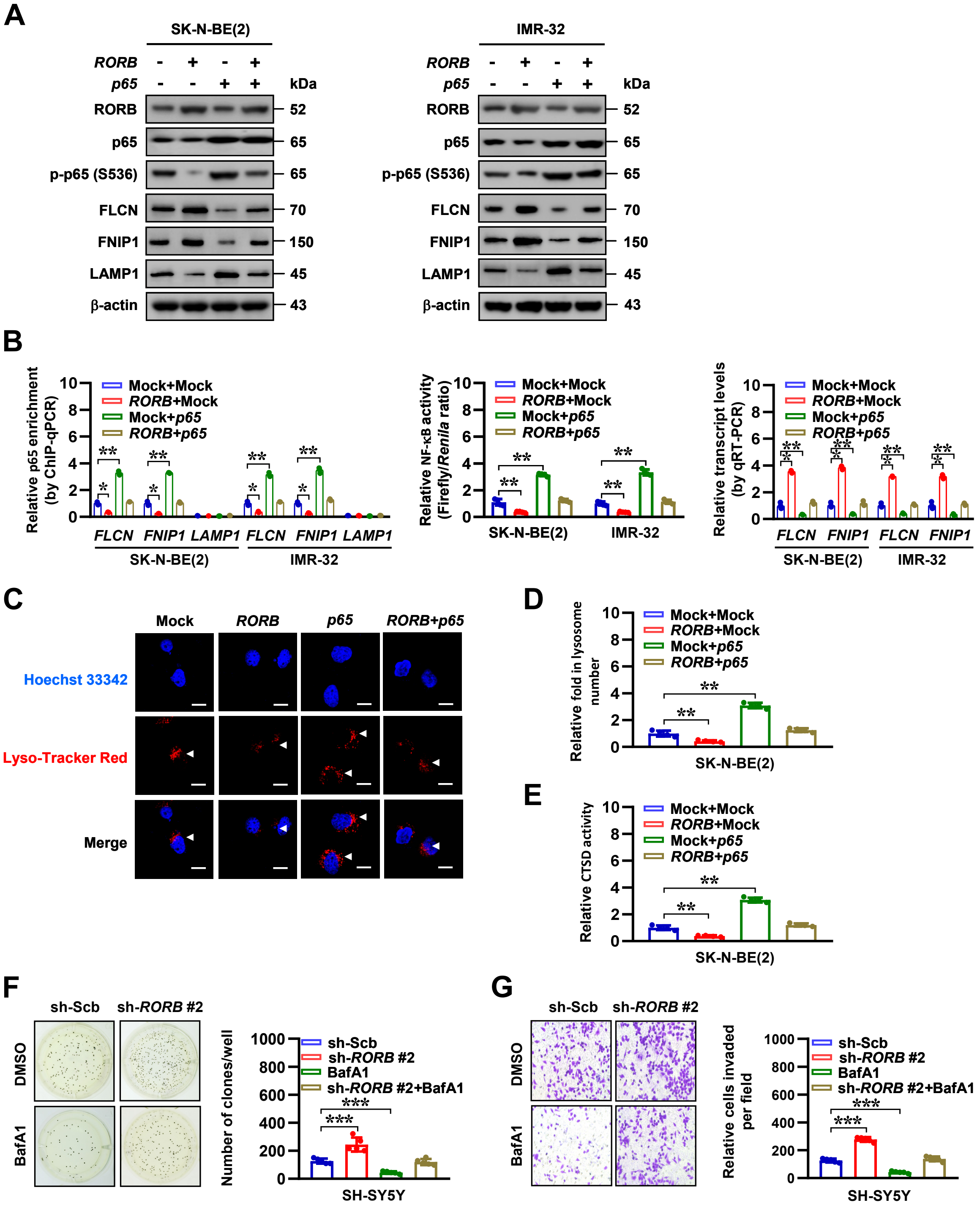

Figure S6. ***RORB* facilitates the expression of *FLCN* and *FNIP1* via repressing NF-κB signaling in NB cells. A**) Western blot assay revealing the levels of RORB, phosphorated or non-phosphorated p65, FLCN, FNIP1, and LAMP1 in SK-N-BE(2) and IMR-32 cells stably transfected with empty vector (mock) or *RORB*, and those co-transfected with *p65*. **B**) ChIP-qPCR (normalized to input, *n*=3), dual-luciferase (*n*=4), and real-time qRT-PCR (normalized to *β-actin*, *n*=4) assays indicating the enrichment or activity of p65 and transcript levels of *FLCN* or *FNIP1* in SK-N-BE(2) and IMR-32 cells stably transfected with mock or *RORB*, and those co-transfected with *p65* (*n*=4). **C** and **D**) Representive images (C) and quantification (D) of fluroscence assay showing Lyso-Tracker Red-positive lysosomes (arrowheads) within SK-N-BE(2) cells stably transfected with mock or *RORB*, and those co-transfected with *p65* (*n*=4). Scale bars: 10 μm. **E**) The CTSD activity in SK-N-BE(2) cells stably transfected with mock or *RORB*, and those co-transfected with *p65* (*n*=4). **F** and **G**) Representive images (left panel) and quantification (right panel) of soft agar (F) and matrigel invasion (G) assays indicating the growth and invasion capabilities of SH-SY5Y stably transfected with scramble shRNA (sh-Scb) or sh-*RORB* #2, and those treated with dimethyl sulfoxide (DMSO) or Bafilomycin A1 (BafA1, 5 nmol·L^{-1}, *n*=5). One-way ANOVA compared the difference in **B** and **D-G**. Data are shown as mean ± s.e.m. (error bars); *, P<0.05; **, P<0.01, ***, P<0.001.

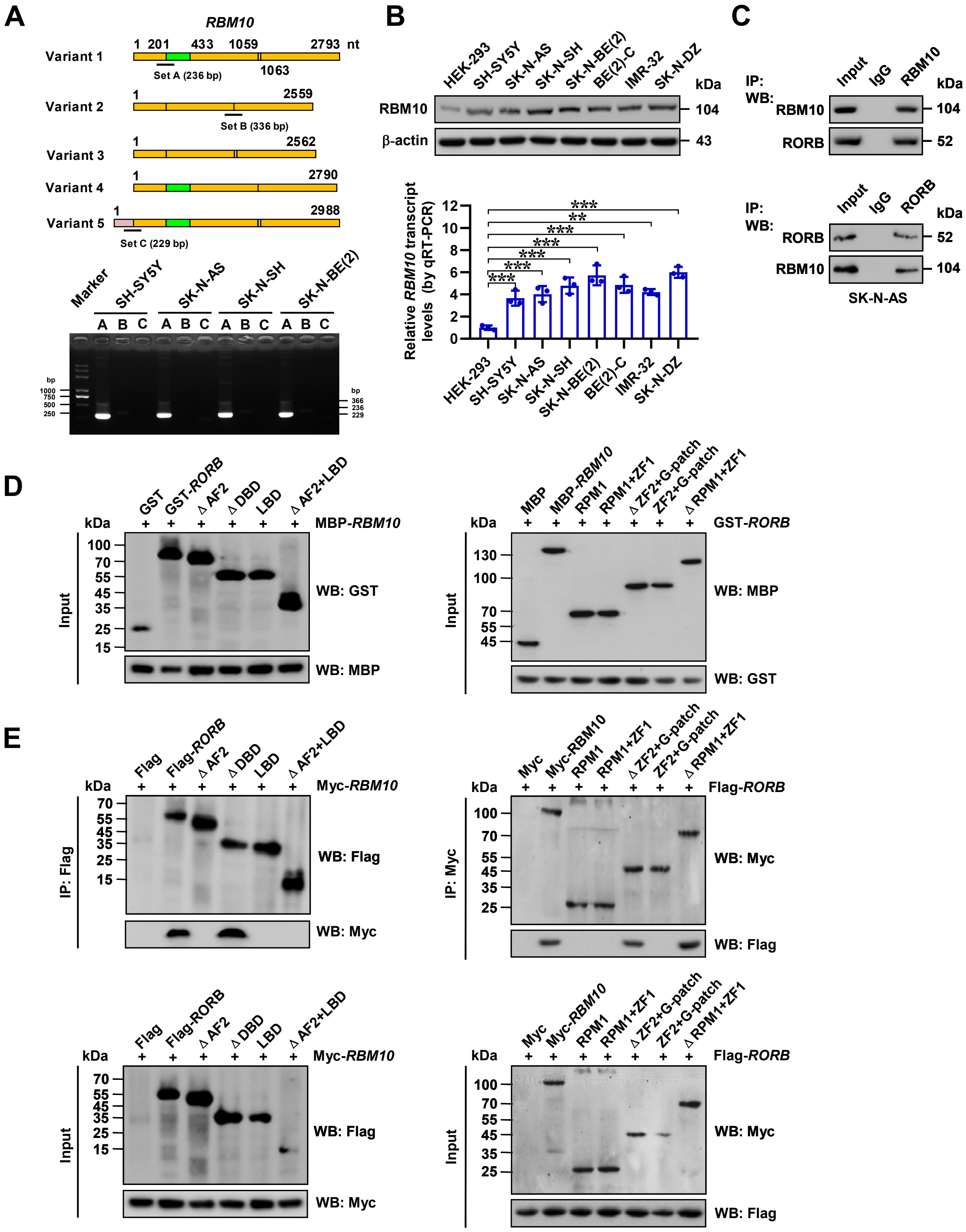

Figure S7. RBM10 directly interacts with RORB protein. A) RT-PCR assay with specific primer sets revealing the existence of *RBM10* variants in cultured NB cell lines. **B**) Western blot (upper panel) and real-time qRT-PCR (lower panel, normalized to *β-actin*, *n*=3) assays showing the protein and transcript levels of *RBM10* in NB cell lines and HEK-293 cells. **C**) Co-IP and western blot assays revealing endogenous interaction of RBM10 and RORB in SK-N-AS cells, with IgG as a negative control. **D**) Western blot assay showing the input group of recombinant GST-tagged RORB and MBP-tagged RBM10 truncation proteins. **E**) Co-IP and western blot assays indicating the interaction of RORB and RBM10 in SK-N-BE(2) cells transfected with Flag-tagged *RORB* and Myc-tagged *RBM10* truncation constructs. One-way ANOVA compared the difference in **B**. Data are shown as mean ± s.e.m. (error bars); ***, P<0.001.

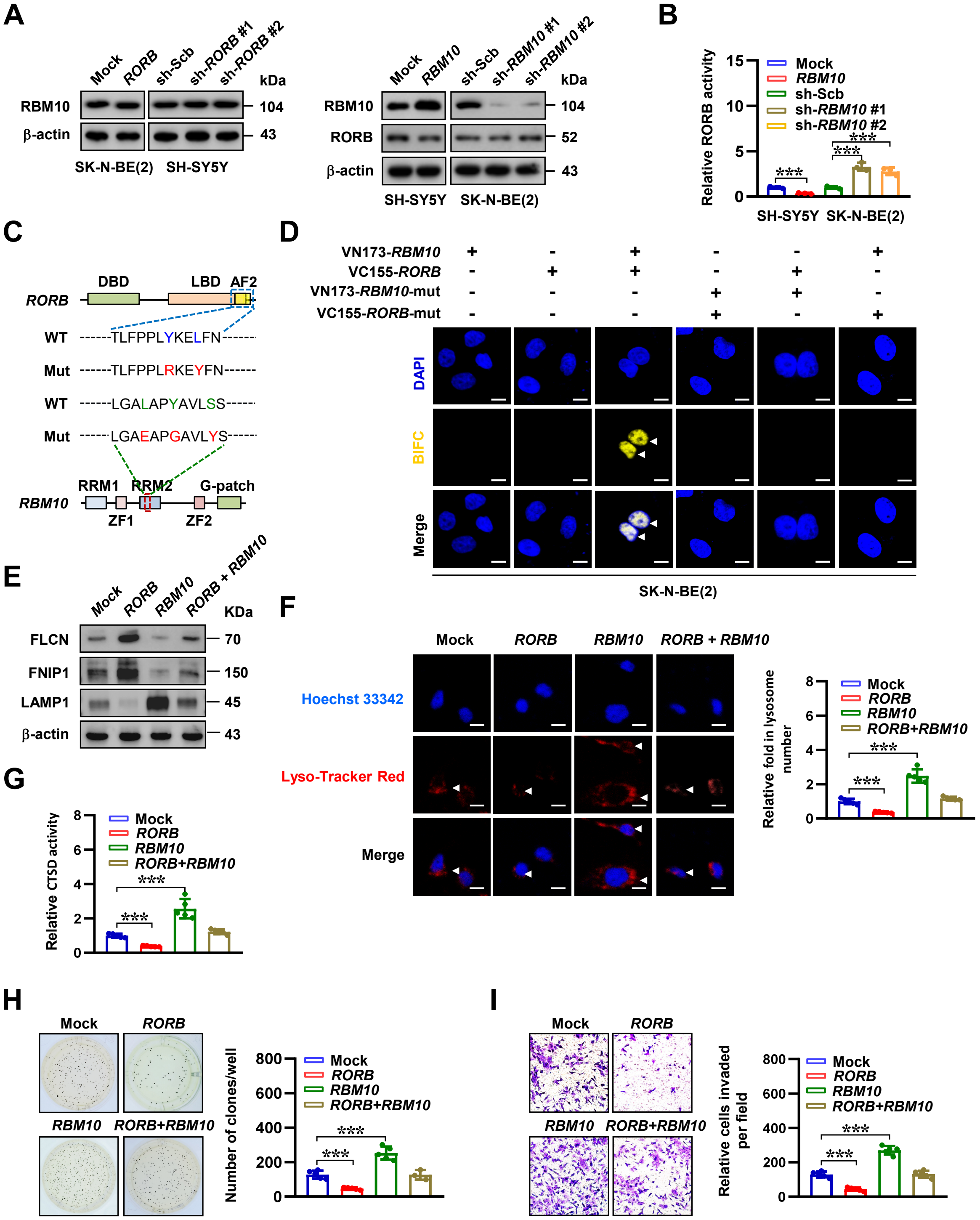

Figure S8. ***RBM10*** **promotes growth and aggressiveness of NB cells via repressing RORB activity. A)** Western blot assay showing the protein levels of RBM10 and RORB in SK-N-BE(2) and SH-SY5Y cells stably transfected with empty vector (mock), *RORB*, *RBM10*, scramble shRNA (sh-Scb), sh-*RORB* #1, sh-*RORB* #2, sh-*RBM10* #1, or sh-*RBM10* #2. **B)** Dual-luciferase reporter assay indicated the activity of RORB in SH-SY5Y and SK-N-BE(2) cells stably transfected with mock, *RBM10*, sh-Scb, sh-*RBM10* #1, or sh-*RBM10* #2 (*n*=3). **C** and **D)** Confocal images of BiFC assay (D) indicating direct interaction between RBM10 and RORB (arrowheads) in SK-N-BE(2) cells co-transfected with wild type (WT) or mutant (Mut) *RBM10* and *RORB* constructs as indicated (C). Scale bars: 10 μm. **E)** Western blot assay revealed the expression levels of FLCN, FNIP1, and LAMP1 in SK-N-BE(2) cells stably transfected with mock, *RORB*, or *RBM10*. **F)** Representive images (left panel) and quantification (right panel) of fluroscence observation showing Lyso-Tracker Red-positive lysosomes (arrowheads) within SK-N-BE(2) stably transfected with mock, *RORB*, or *RBM10* (*n*=5). Scale bars: 10 μm. **G)** The CTSD activity in SK-N-BE(2) cells stably transfected with mock, *RORB*, or *RBM10* (*n*=5). **H** and **I)** Representive images (left panel) and quantification (right panel) of soft agar (H) and matrigel invasion (I) assays indicating the growth and invasion capabilities of SK-N-BE(2) cells stably transfected with mock, *RORB*, or *RBM10* (*n*=5). One-way ANOVA compared the difference in **B** and **F-I**. Data are shown as mean ± s.e.m. (error bars); ***, P<0.001.

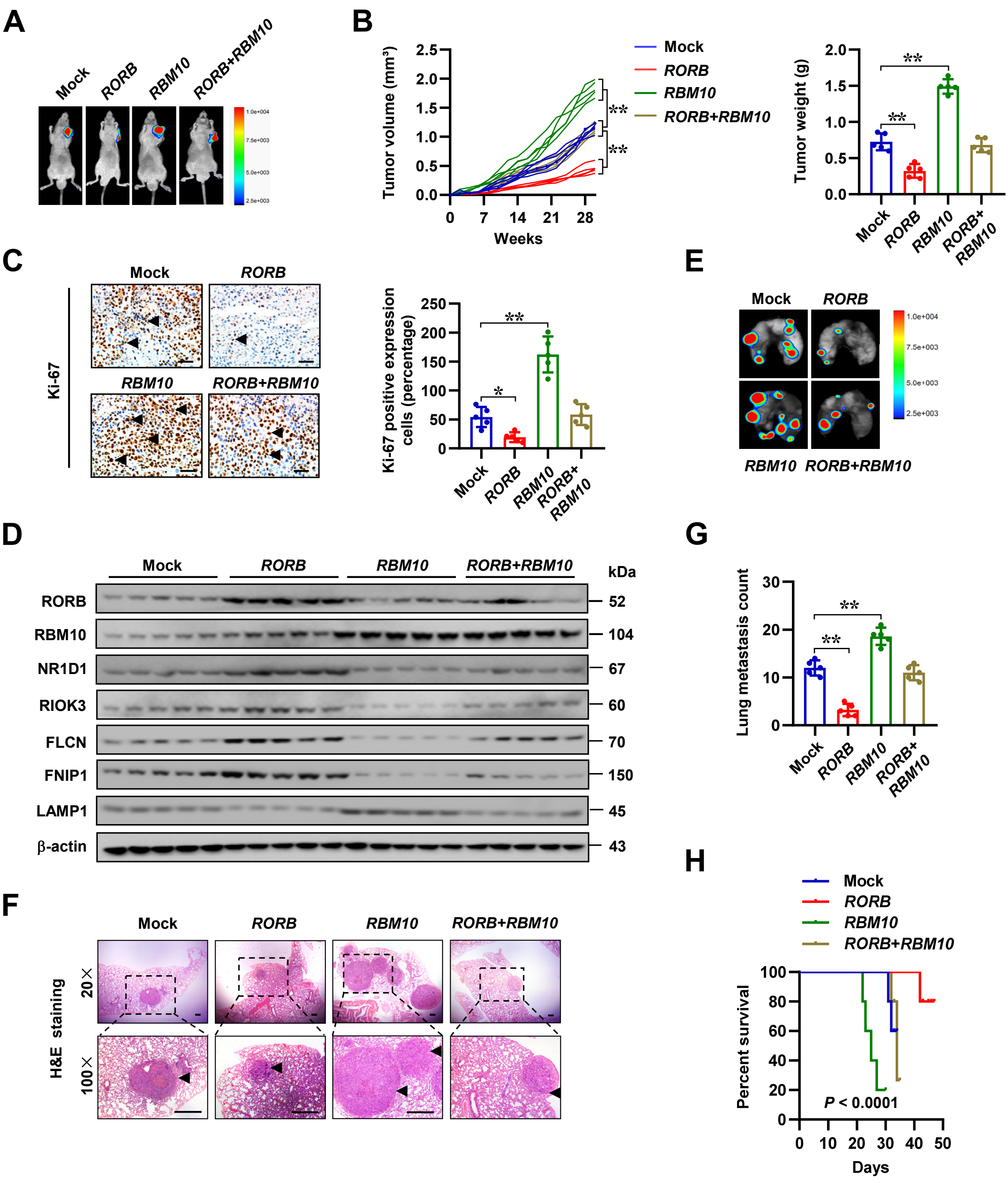

Figure S9. ***RBM10*** **promotes NB progression via repressing RORB activity. A**) Representative images of subcutaneous xenograft tumors formed by SK-N-BE(2) cells stably transfected with empty vector (mock), *RORB*, or *RBM10* in nude mice (*n*=5 per group). **B**) Growth curve and weight of subcutaneous xenograft tumors in nude mice formed by SK-N-BE(2) cells stably transfected with mock, *RORB*, or *RBM10* in nude mice (*n*=5 per group). **C**) Representative images (left panel) and quantification (right panel) of Ki-67 immunostaining (arrowheads) within subcutaneous xenograft tumors formed by SK-N-BE(2) cells stably transfected with mock, *RORB*, or *RBM10* in nude mice (*n*=5 per group). **D**) Western blot assay showing the levels of RORB, RBM10, NR1D1, RIOK3, FLCN, FNIP1, or LAMP1 in subcutaneous xenograft tumors in nude mice formed by SK-N-BE(2) cells stably transfected with mock, *RORB*, or *RBM10* in nude mice (*n*=5 per group). **E-G**) *In vivo* imaging (E), HE staining (F, arrowheads), and quantification (G) of lung metastasis in nude mice treated with tail vein injection of SK-N-BE(2) cells stably transfected with mock, *RORB*, or *RBM10* (*n*=5 per group). Scale bar, 100 μm. **H**) Kaplan-Meier curves of nude mice treated with tail vein injection of SK-N-BE(2) cells stably transfected with mock, *RORB*, or *RBM10* (*n*=5 per group). One-way ANOVA compared the difference in **B**, **C**, and **G**. Log-rank test for survival comparison in **H**. Data are shown as mean ± s.e.m. (error bars); *, P<0.05; **, P<0.01.

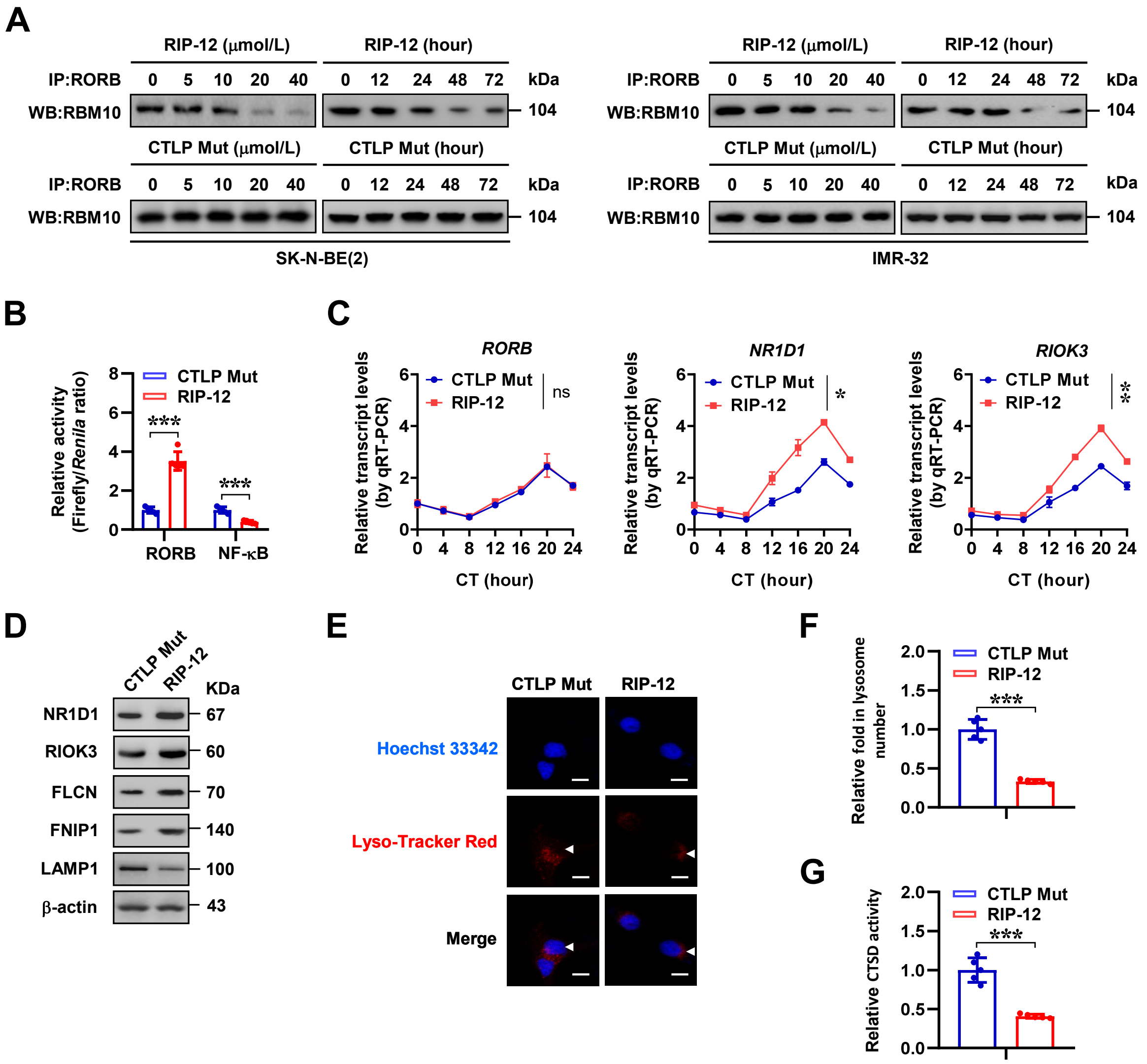

Figure S10. RIP-12 inhibits lysosomal biogenesis of NB cells via blocking RBM10-RORB interaction. A) Co-IP and western blot assays indicating the interaction between RBM10 and RORB in SK-N-BE(2) and IMR-32 cells treated with CTLP Mut or RIP-12 for 24 hours or different duration (10 μmol·L^{-1}) as indicated. **B**) Dual-luciferase reporter assay showing the activity of RORB and NF-κB in IMR-32 cells treated with CTLP Mut or RIP-12 (10 μmol·L^{-1}, *n*=5). **C**) Real-time qRT-PCR (normalized to *β-actin*, *n*=4) assay revealing the circadian transcript levels of *RORB*, *NR1D1* and *RIOK3* in IMR-32 cells treated with CTLP Mut or RIP-12 (10 μmol·L^{-1}). **D**) Western blot assay showing the levels of NR1D1, RIOK3, FLCN, FNIP1, or LAMP1 in IMR-32 cells treated with CTLP Mut or RIP-12 (10 μmol·L^{-1}). **E** and **F**) Representive images (E) and quantification (F, *n*=5) of fluroscence observation showing Lyso-Tracker Red-positive lysosomes (arrowheads) within IMR-32 cells treated with CTLP Mut or RIP-12 (10 μmol·L^{-1}). Scale bars: 10 μm. **G**) The CTSD activity in IMR-32 cells treated with CTLP Mut or RIP-12 (10 μmol·L^{-1}, *n*=5). Student's *t*-test or one-way ANOVA compared the difference in **B**, **C**, **F** and **G**. Data are shown as mean ± s.e.m. (error bars); *, *P*<0.05; **, *P*<0.01; ***, *P*<0.001; ns, non-significant.

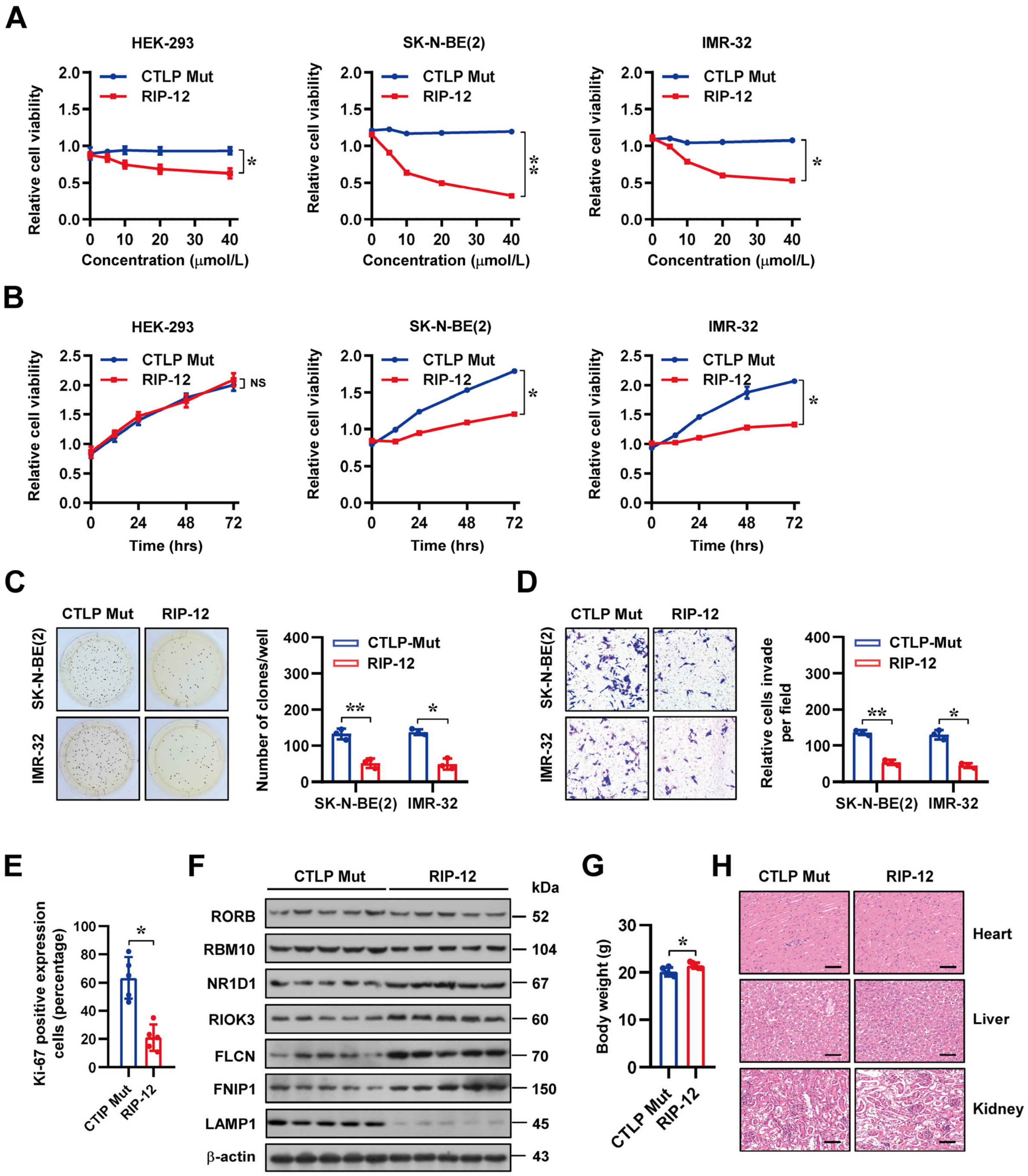

Figure S11. RIP-12 inhibits the growth, invasion, and metastasis of NB cells. A and **B**) MTT colorimetric assay indicating the viability of HEK-293, SK-N-BE(2), and IMR-32 cells treated with CTLP Mut or RIP-12 for 24 hours or different duration (10 μmol · L^{-1}) as indicated (n=5). **C** and **D**) Representative images (left panel) and quantification (right panel) of soft agar (C) and matrigel invasion (D) assays showing the growth and invasion of SK-N-BE(2) and IMR-32 cells treated with CTLP or RIP-12 (10 μmol · L^{-1}, n=3). **E**) Body weight of nude mice receiving subcutaneous injection of IMR-32 cells and tail vein injection of administration of CTLP Mut or RIP-12 (3 mg·kg^{-1}, n=5 per group). **F**) Western blot assay revealing the expression of RORB, RBM10, NR1D1, RIOK2, FLCN, FNIP1, or LAMP1 in subcutaneous xenograft tumors formed by IMR-32 cells treated with CTLP Mut or RIP-12 (3 mg·kg^{-1}, n=5 per group). **G**) Quantification of Ki-67 immunostaining within subcutaneous xenograft tumors formed by IMR-32 cells treated with CTLP Mut or RIP-12 (3 mg·kg^{-1}, n=5 per group). **H**) HE staining showing the morphological changes of hear, liver, and kidney tissues of nude mice receiving subcutaneous injection of IMR-32 cells and tail vein injection of administration of CTLP Mut or RIP-12 (3 mg·kg^{-1}, n=5 per group). One-way ANOVA or Student's t-test compared the difference in **A**-**E** and **G**. Data are shown as mean ± s.e.m. (error bars); *, P<0.05; **, P<0.01; ns, non-significant.

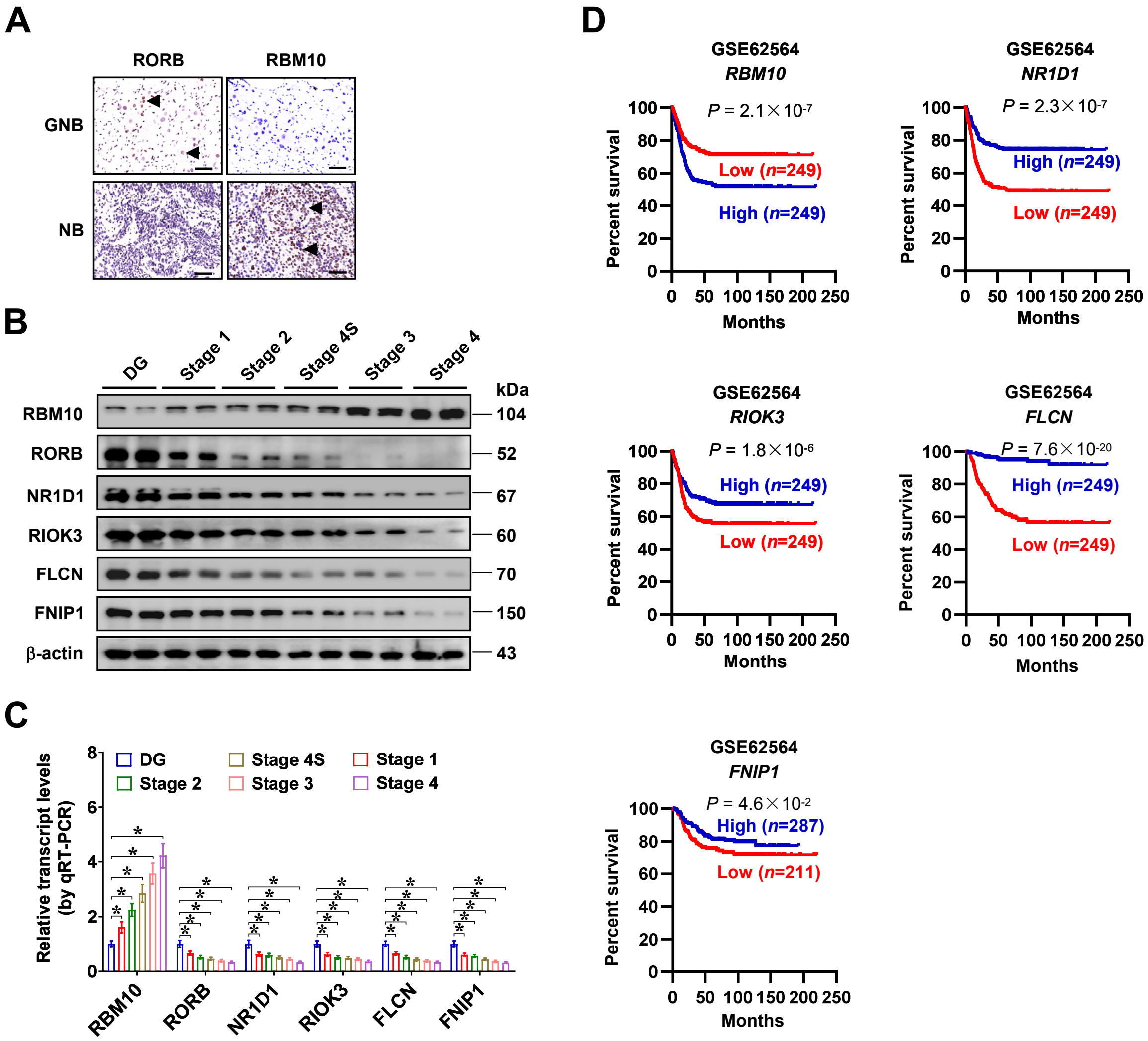

Figure S12. *RBM10*/*RORB* axis and target genes are associated with prognosis of NB patients. A) Immunohistochemistry indicating the expression level of RORB and RBM10 (arrowheads) in GNB and NB tumor tissues. **B** and **C**) Western blot (B) and real-time qRT-PCR (C, normalized to *β-actin*) assays showing the expression levels of *RORB*, *RBM10*, *NR1D1*, *RIOK3*, *FLCN*, or *FNIP1* in normal dorsal root ganglia (DG, n=3) and NB tissues of various stages (n=15). **D**) Kaplan–Meier curves indicating overall survival of 498 well-defined NB cases (GSE62564) with high or low expression of *RBM10* (cutoff value=6.07), *NR1D1* (cutoff value=4.20), *RIOK3* (cutoff value=6.09), *FLCN* (cutoff value=5.61), or *FNIP1* (cutoff value=4.89). One-way ANOVA compared the difference in **C**. Log-rank test for survival comparison in **D**. Data are shown as mean ± s.e.m. (error bars); *, P<0.05.

Table S1 Association of transcription factor expression with NB patients' overall survival in three independent NB cohorts

GEO dataset	Transcription factors (TFs)		
	**RORB**	**ZEB2**	**L3MBTL4**
GSE85047	low is worse; P=9.7×10^{-4}	low is worse; P=1.3×10^{-4}	high is worse; P=6.9×10^{-4}
GSE45547	low is worse; P=7.0×10^{-6}	low is worse; P=6.5×10^{-6}	low is worse; P=3.1×10^{-2}
GSE62564	low is worse; P=2.0×10^{-2}	high is worse; P=7.6×10^{-5}	high is worse; P=8.5×10^{-2}

TFs, transcription factors; RORB, RAR related orphan receptor B; ZEB2, zinc finger E-box binding homeobox 2; L3MBTL4, L3MBTL histone methyl-lysine binding protein 4.

Table S2 Identification of RORB-binding proteins in NB cells by mass spectrometry

SH-SY5Y		SK-N-AS		
ADGRL2	RAVER1	ACBD5	LRPPRC	RPL6
ANKRD40	RBM10	ACP1	LYAR	RPL7
ARID1B	RPS27	ACTBL2	MAGOH	RPL8
ATP1A1	SDE2	ACTL6A	MCM3	RPN2
ATP1B3	SELENOF	ACTR3	MRE11	RPS11
ATP2A2	SLC1A5	AKAP8	MRPL39	RPS15A
ATP5F1D	SLC7A5	ANXA5	NCSTN	RPS16
BAP18	SLIRP	ATL3	NDUFA2	RPS21
BSG	SRSF2	ATP2A2	NEXN	RPS9
BTF3	STUB1	BASP1	NME1	RRP8
CLTA	TCOF1	CAPNS1	NT5E	SCAF8
COPE	THOC3	CAT	NUDT21	SCAMP3
CTNND1	TMED10	CCAR2	NUP50	SEC22B
DDX6	TNRC6B	CHD4	NUP98	SF3B6
DNAJA2	TP53	CISD1	PBXIP1	SFXN3
DYNLT1	TPM3	CLNS1A	PCBP2	SLTM
EEF1E1	TUBB2A	CLPX	PFDN6	SMC1A
EIF3I	UQCRB	COX4I1	PMVK	SMC3
EIF5A	VAPA	COX6C	PPIL3	SND1
GAPDH	XPO1	DDX3X	PRDX6	SVIL
GEN1	ZRANB2	DHFR	PRKAR2A	SYNM
H2AFX		DNAJB12	PSMC1	TARDBP
HSPH1		EEF2	PSMC3	TECR
IKBIP		EIF2S1	PSMD4	TIMM50
LSM3		EMC8	PTCD3	TOMM40
MAP7D1		ERGIC1	QKI	TOR1AIP1
MRE11		ESYT1	RAB10	TPM1
MRPL12		ETHE1	RAB14	TPM4
NAT10		FAM162A	RAB21	TRIM21
NDUFS1		FTH1	RAB8A	TUBG1
NME2		FUBP1	RAB9A	VTN
NSMCE1		FUBP3	RAN	ZNF385A
OTX1		GNG12	RBBP6	ZNF638
PHGDH		GPRC5A	RBM10	
PKM		HADHA	RFC1	
POLR2E		HDAC1	RING1	
PPIE		HEXIM1	RORB	
PPP1CB		HSP90AA1	RPL21	
R3HDM1		HSP90AB1	RPL35	
RAB7A		JUND	RPL4	

Table S3 Primer sets used for RT-PCR, qPCR, and ChIP assays

Primer set	Primers	Sequence	Product size (bp)	Application
RORB variant 1	Forward	5'-ATGCGAGCACAAATTGAAGTG -3'	183	RT-PCR
	Reverse	5'-ACGGTTTCTGTTCGTTCTGTC-3'		
RORB variant 2	Forward	5'-AAACTAAAGCTGACGCCACTG-3'	494	RT-PCR
	Reverse	5'-GGAATCGACGTTGTAATAACCC -3'		
RORB	Forward	5'-CCTATCTATGACCTCACATCCGTAC-3'	240	qPCR
	Reverse	5'-TTGTTGCCACAGTGCTTCCCT-3'		
RBM10 set A	Forward	5'-GGGCAAGCATGACTATGACG-3'	236	RT-PCR
	Reverse	5'-CGATGTTACTGGCCTTCTCCT-3'		
RBM10 set B	Forward	5'-CCACCATCGAGGCAGCCC-3'	366	RT-PCR
	Reverse	5'-CCCCTTTGGTTCCAGTGA-3'		
RBM10 set C	Forward	5'-CCCCGAAGGCCCTATCAG-3'	229	RT-PCR
	Reverse	5'-AGGAATCCTCCGCACTCT-3'		
RBM10	Forward	5'-AGAAGGCCAGTAACATCGTCA-3'	194	qPCR
	Reverse	5'-TTCCATCCATCGTGTAGCG-3'		
NR1D1	Forward	5’-CCCCAATGACAACAACACCT-3’	244	qPCR
	Reverse	5’-CATAGGACATGCCAGCAGAAC-3’		
RIOK3	Forward	5’-ATGTTGGAGACAATCACTGGCT-3’	235	qPCR
	Reverse	5’-TGCGGATGATCTTACGTGGA-3’		
FLCN	Forward	5’-CTACACCAGAGGAACGGCAACG-3’	102	qPCR
	Reverse	5’-CTTCAGGAGCCAGGCAAAGGAG-3’		
FNIP1	Forward	5’-CCCCAAACTCCTCACTTACC-3’	147	qPCR
	Reverse	5’-TAGGGCCACAGCTTTCATCT-3’		
ACTB	Forward	5'-TGCCCATCTACGAGGGGTATG-3'	156	qPCR,
	Reverse	5'-TCTCCTTAATGTCACGCACGATTT-3'		
NR1D1	Forward	5'-TTTAGGACCTCAAAGCACTTTC-3’	228	ChIP
	Reverse	5'-GAAGGAAAGGAAATGTGACCC-3’		
RIOK3	Forward	5'-AAAACTCTTGGACCTGAACCTT-3’	172	ChIP
	Reverse	5'-CATTGGTGAGATGGGCTGTAA-3’		
FLCN	Forward	5'-TAAGTTTCAGCATACTCCCG-3’	129	ChIP
	Reverse	5'-GACAATTACCCTTTTCCACC-3’		
FNIP1	Forward	5’-TGCTCGAGGTCGTCTTTGCG-3’	228	ChIP
	Reverse	5’-CAGAGTCTGGCGGCTCCTGG-3’		

RORB, RAR related orphan receptor B; RBM10, RNA binding motif protein 10; NR1D1, nuclear receptor subfamily 1 group D member 1; RIOK3, RIO kinase 3; FLCN, folliculin; FNIP1, folliculin interacting protein 1; ACTB, beta-actin; ChIP, chromatin immunoprecipitation.

Table S4 Oligonucleotide sets used for construct preparation

Oligo Set	Sequences
pGL3-RORB-Luc	5'-TGCCAGAACATTTCTCTATCGATAGGTACCAGTAGACATCTCCCAGCCTCAA-3' (sense);<br>5'-CTTTACCAACAGTACCGGAATGCCAAGCTTATGACATCTGTGCCTGTTAGCG-3' (antisense)
pCMV-3Tag-1A-RORB (1-1380)	5'-CCGGAATTCATGCGAGCACAAATTGAAGT-3' (sense);<br>5'-CCGCTCGAGTCATTTGCAGCCGGTGGCAC-3' (antisense)
pCMV-3Tag-1A-RORB (1-1332)	5'-CCGGAATTCATGCGAGCACAAATTGAAGT-3' (sense);<br>5'-CCGCTCGAGCGGAGGAAACAGTGTATTCA-3' (antisense)
pCMV-3Tag-1A-RORB (631-1380)	5'-CCGGAATTCGAAATCGACCGAATTGCACA-3' (sense);<br>5'-CCGCTCGAGTCATTTGCAGCCGGTGGCAC-3' (antisense)
pCMV-3Tag-1A-RORB (1-246)	5'-CCGGAATTCATGCGAGCACAAATTGAAGT-3' (sense);<br>5'-CCGCTCGAGAAACTTCACAGCATCTCTTG-3' (antisense)
pCMV-3Tag-1A-RORB (631-1347)	5'-CCGGAATTCGAAATCGACCGAATTGCACA-3' (sense);<br>5'-CCGCTCGAGGAGCTCCTTGTATAACGGAG-3' (antisense)
pGEX-6P-1-RORB (1-1380)	5'-CCGGAATTCATGCGAGCACAAATTGAAGT-3' (sense);<br>5'-CCGCTCGAGTCATTTGCAGCCGGTGGCAC-3' (antisense)
pGEX-6P-1-RORB (1-1332)	5'-CCGGAATTCATGCGAGCACAAATTGAAGT-3' (sense);<br>5'-CCGCTCGAGCGGAGGAAACAGTGTATTCA-3' (antisense)
pGEX-6P-1-RORB (631-1380)	5'-CCGGAATTCGAAATCGACCGAATTGCACA-3' (sense);<br>5'-CCGCTCGAGTCATTTGCAGCCGGTGGCAC-3' (antisense)
pGEX-6P-1-RORB (1-246)	5'-CCGGAATTCATGCGAGCACAAATTGAAGT-3' (sense);<br>5'-CCGCTCGAGAAACTTCACAGCATCTCTTG-3' (antisense)
pGEX-6P-1-RORB (631-1347)	5'-CCGGAATTCGAAATCGACCGAATTGCACA-3' (sense);<br>5'-CCGCTCGAGGAGCTCCTTGTATAACGGAG-3' (antisense)
pBiFC-VC155-RORB	5'-ATGGCCATGGAGGCCCGAATTCGGATGCGAGCACAAATTGAAGT-3' (sense)<br>5'-TTTTGCACGCCGGACGGGTACCTTTGCAGCCGGTGGCACAGT-3' (antisense)
pBiFC-VC155-RORB-mut	5'-CTGTTTCCTCTGATGTACAAGGAGCGCGCTAATCCTGACTGTGCCACCGGCTGCAAAGGT-3' (sense)<br>5'-AGTCAGGATTAGCGCGCTCCTTGTACATCAGAGGAAACAGTGTATTCACTATCTCTGGAT-3' (antisense)
Lenti-CV186-RORB	5'-CTTGGGCTGCAGGTCGACTCTAGAGGATCCATGGAGTATGAAAGACGTGGTGGT-3' (sense);<br>5'-GTCATCGTCATCCTTGTAGTCCATACCGGTCTGGGCCTCGTTGAAGCGGGTCAC-3' (antisense)
pCMV-N-MYC-RBM10 (1-2793)	5'-TCTGAAGAGGATCTGAGCCCGGGCGGATCCATGGAGTATGAAAGACGTGGTGGT-3' (sense);<br>5'-GGTACCGGGCCCACTAGTTCTAGACTCGAGTCACTGGGCCTCGTTGAAGCGGGT-3' (antisense)
pCMV-N-MYC-RBM10 (1-627)	5'-TCTGAAGAGGATCTGAGCCCGGGCGGATCCATGGAGTATGAAAGACGTGGTGGT-3' (sense);<br>5'-GGTACCGGGCCCACTAGTTCTAGACTCGAGGGGGTCACTGTAGTGCATCGACAC-3' (antisense)
pCMV-N-MYC-RBM10 (1-726)	5'-TCTGAAGAGGATCTGAGCCCGGGCGGATCCATGGAGTATGAAAGACGTGGTGGT-3' (sense);<br>5'-GGTACCGGGCCCACTAGTTCTAGACTCGAGCTCTGACTTGGGCACGCCACATTT-3' (antisense)
pCMV-N-MYC-RBM10 (1-1350)	5'-TCTGAAGAGGATCTGAGCCCGGGCGGATCCATGGAGTATGAAAGACGTGGTGGT-3' (sense);<br>5'-GGTACCGGGCCCACTAGTTCTAGACTCGAGTGTGCCCTGGCTGTTGCCATAGCC-3' (antisense)
pCMV-N-MYC-RBM10 (1438-2793)	5'-TCTGAAGAGGATCTGAGCCCGGGCGGATCCCCCGAGGCCTCCCTAGAGCCTGGG-3' (sense);<br>5'-GGTACCGGGCCCACTAGTTCTAGACTCGAGTCACTGGGCCTCGTTGAAGCGGGT-3' (antisense)
pCMV-N-MYC-RBM10 (898-2793)	5'-TCTGAAGAGGATCTGAGCCCGGGCGGATCCGACACCATCATTTTGCGCAACCTG -3' (sense);<br>5'-GGTACCGGGCCCACTAGTTCTAGACTCGAGTCACTGGGCCTCGTTGAAGCGGGT-3' (antisense)
pMAL-c4X-RBM10 (1-2793)	5'-ATCGAGGGAAGGATTTCAGAATTCGGATCCATGGAGTATGAAAGACGTGGTGGT-3' (sense);<br>5'-CGTTGTAAAACGACGGCCAGTGCCAAGCTTTCACTGGGCCTCGTTGAAGCGGGT-3' (antisense)
pMAL-c4X-RBM10 (1-627)	5'-ATCGAGGGAAGGATTTCAGAATTCGGATCCATGGAGTATGAAAGACGTGGTGGT-3' (sense);<br>5'-CGTTGTAAAACGACGGCCAGTGCCAAGCTTTCACTGGGCCTCGTTGAAGCGGGT-3' (antisense)
pMAL-c4X-RBM10 (1-726)	5'-ATCGAGGGAAGGATTTCAGAATTCGGATCCATGGAGTATGAAAGACGTGGTGGT-3' (sense);<br>5'-CGTTGTAAAACGACGGCCAGTGCCAAGCTTCTCTGACTTGGGCACGCCACATTT-3' (antisense)
pMAL-c4X-RBM10 (1-1350)	5'-ATCGAGGGAAGGATTTCAGAATTCGGATCCATGGAGTATGAAAGACGTGGTGGT-3' (sense);<br>5'-CGTTGTAAAACGACGGCCAGTGCCAAGCTTTGTGCCCTGGCTGTTGCCATAGCC-3' (antisense)
pMAL-c4X-RBM10 (1438-2793)	5'-ATCGAGGGAAGGATTTCAGAATTCGGATCCCCCGAGGCCTCCCTAGAGCCTGGG-3' (sense);<br>5'-CGTTGTAAAACGACGGCCAGTGCCAAGCTTTCACTGGGCCTCGTTGAAGCGGGT-3' (antisense)
pMAL-c4X-RBM10 (898-2793)	5'-ATCGAGGGAAGGATTTCAGAATTCGGATCCGACACCATCATTTTGCGCAACCTG-3' (sense);<br>5'-CGTTGTAAAACGACGGCCAGTGCCAAGCTTTCACTGGGCCTCGTTGAAGCGGGT-3' (antisense)
pBiFC-VN173-RBM10	5'-ACAAGCTTGCGGCCGCGAATTCCATGGAGTATGAAAGACGTGG-3' (sense)<br>5'-TCTTCTAGAGTCGACTGGTACCCCCTGGGCCTCGTTGAAGCGGG-3' (antisense)
pBiFC-VN173-RBM10-mut	5'-CCTGGCACCCAACGCGGTGAAGTCCTCCTCCAACGTGCGCGTCATAAAGGAC-3' (sense)<br>5'-TGGAGGAGGACTTCACCGCGTTGGGTGCCAGGGCCCCCAGGATGGAATCCAT-3' (antisense)
Lenti-CV186-RBM10	5'-CTTGGGCTGCAGGTCGACTCTAGAGGATCCATGGAGTATGAAAGACGTGGTGGT-3' (sense);<br>5'-GTCATCGTCATCCTTGTAGTCCATACCGGTCTGGGCCTCGTTGAAGCGGGTCAC-3' (antisense)
pGL3-NR1D1-Luc	5'-TGCCAGAACATTTCTCTATCGATAGGTACCAGATCTGTTATTACTCATGGGACA-3' (sense);<br>5'-CTTTACCAACAGTACCGGAATGCCAAGCTTAACCAGGAAGTAAGTAGGTGATGG-3' (antisense)
pGL3-RIOK3-Luc	5'-TGCCAGAACATTTCTCTATCGATAGGTACCCAGGAGAATTGCTGCTTGAA-3' (sense)<br>5'-CTTTACCAACAGTACCGGAATGCCAAGCTTATCGGTTTGGGAACTGGACT-3' (antisense)
pET28a-mCherry-RORB	5'-TGGACAGCAAATGGGTCGCGGAATGCGAGCACAAATTGAAGT-3' (sense)<br>5'-GCCCTTGCTCACCATGGATCCTTTGCAGCCGGTGGCACAGT-3' (antisense)
pET28a-EGFP-RBM10	5'-CCGGAATTCATGGAGTATGAAAGACGTGG-3' (sense)<br>5'-CCCAAGCTTCTGGGCCTCGTTGAAGCGGG-3' (antisense)

RORB, RAR related orphan receptor B; RBM10, RNA binding motif protein 10; NR1D1, nuclear receptor subfamily 1 group D member 1; RIOK3, RIO kinase 3.

Table S5 Oligonucleotide sets used for preparation of short hairpin RNAs

Oligo Set	Sequences
sh-Scb	5'-AGGGATACAAGCATATACCACTCGAGTGGTATATGCTTGTATCCCTC-3' (sense);
	5'-GAGGGATACAAGCATATACCACTCGAGTGGTATATGCTTGTATCCCT-3' (antisense)
sh-RORB #1	5'-CCGGTCGGCCTGAGCAACCTGAACCTCGAGGTTCAGGTTGCTCAGGCCGTTTTTG-3' (Sense)
	5'-GATCCAAAAACGGCCTGAGCAACCTGAACCTCGAGGTTCAGGTTGCTCAGGCCGA -3' (Antisense)
sh-RORB #2	5'-CCGGTCAGATAAAGCAAGAACCTACTCGAGTAGGTTCTTGCTTTATCTGTTTTTG-3' (Sense)
	5'-GATCCAAAAACAGATAAAGCAAGAACCTACTCGAGTAGGTTCTTGCTTTATCTGA-3' (Antisense)
sh-RBM10 #1	5'-CCGGTGACATGGACTACCGTTCATATCTCGAGATATGAACGGTAGTCCATGTCTTTTTG-3' (Sense)
	5'-GATCCAAAAAGACATGGACTACCGTTCATATCTCGAGATATGAACGGTAGTCCATGTCA-3' (Antisense)
sh-RBM10 #2	5'-CCGGTCTTCGCCTTCGTCGAGTTTAGCTCGAGCTAAACTCGACGAAGGCGAAGTTTTTG-3' (Sense)
	5'-GATCCAAAAACTTCGCCTTCGTCGAGTTTAGCTCGAGCTAAACTCGACGAAGGCGAAGA-3' (Antisense)
sh-NR1D1 #1	5'-CCGGTCCAGCCCTGAATCCCTCTATACTCGAGTATAGAGGGATTCAGGGCTGGTTTTTG-3' (Sense)
	5'-GATCCAAAAACCAGCCCTGAATCCCTCTATACTCGAGTATAGAGGGATTCAGGGCTGGA-3' (Antisense)
sh-NR1D1 #2	5'-CCGGTGCGCTTTGCTTCGTTGTTCAACTCGAGTTGAACAACGAAGCAAAGCGCTTTTTG-3' (Sense)
	5'-GATCCAAAAAGCGCTTTGCTTCGTTGTTCAACTCGAGTTGAACAACGAAGCAAAGCGCA-3' (Antisense)
sh-RIOK3 #1	5'-CCGGTCTGTTGTCTTTCATGCATATGCTCGAGCATATGCATGAAAGACAACAGTTTTTG-3' (Sense)
	5'-GATCCAAAAACTGTTGTCTTTCATGCATATGCTCGAGCATATGCATGAAAGACAACAGA-3' (Antisense)
sh-RIOK3 #2	5'-CCGGTGCTGACCTCAGTGAGTATAACCTCGAGGTTATACTCACTGAGGTCAGCTTTTTG-3' (Sense)
	5'-GATCCAAAAAGCTGACCTCAGTGAGTATAACCTCGAGGTTATACTCACTGAGGTCAGCA-3' (Antisense)

Scb, scramble; RORB, RAR related orphan receptor B; RBM10, RNA binding motif protein 10; NR1D1, nuclear receptor subfamily 1 group D member 1; RIOK3, RIO kinase 3.

Detailed Experimental Section

Cell lines and culture: Human cell lines were obtained from American Type Culture Collection (ATCC, Rockville, MD), including HEK-293 (CRL-1573), HEK-293T (CRL-3216), SH-SY5Y (CRL-2266), SK-N-AS (CRL-2137), SK-N-SH (HTB-11), SK-N-BE(2) (CRL-2271), BE(2)-C (CRL-2268), IMR-32 (CCL-127), and SK-N-DZ (CRL-2149). All cell lines were authenticated through short tandem repeat (STR) profiling and utilized within six months of resuscitation. Routine mycoplasma screening was performed using the MycoAlert® PLUS Mycoplasma Detection Kit (Takara, Japan), with all cultures testing negative for contamination. Cell lines SK-N-DZ, IMR-32, SK-N-AS, SK-N-SH, HEK293, and HEK-293T were maintained at 37°C under 5% CO_2 in a humid environment using Dulbecco's Modified Eagle's Medium (DMEM; Invitrogen, Carlsbad, CA). Meanwhile, SK-N-BE(2), BE(2)-C, and SH-SY5Y cells were cultured under identical temperature and CO_2 conditions, utilizing either Minimum Essential Medium (MEM)/F12 or DMEM/F12 (Invitrogen) supplemented with 10% fetal bovine serum (FBS; Sigma, St. Louis, MO). To achieve synchronization *in vitro*, cells were incubated with dexamethasone (100 $nmol \cdot L^{-1}$, Sigma, St. Louis, MO) for 60 minutes, and subsequently maintained in complete growth medium. Twenty-four hours after synchronization, cells were harvested at seven sequential time points (4-hour intervals).

***RNA isolation and real-time quantitative RT-PCR (qRT-PCR)*:** Total RNA was extracted using the RNeasy Mini Kit (Qiagen, Hilden, Germany). Reverse transcription was performed with ProtoScript® II First Strand cDNA Synthesis Kit (New England Biolabs, Inc. Ipswich, MA). Quantitative real-time PCR (qPCR) was carried out with SYBR Green PCR Master Mix (Takara) and primers (Table S3). Relative mRNA levels were determined by $2^{-\triangle\triangle Ct}$ method.

***Western blotting*:** Proteins were prepared from tissues or cultured cells using 1× RIPA lysis buffer (Promega, Madison, WI). Western blot analysis was conducted following standard protocols, using antibodies specific against RORB (ab228650), RBM10 (ab72423), NR1D1 (ab174309), RIOK3 (ab241361), phospho-IKKα/β (p-IKKα/β at Ser176/180, ab17943), IKKα (ab32041), IKKβ (ab124957), phospho-IκBα (p-IκBα at Ser32/36, ab133462), IκBα (ab32518), phospho-p65 (p-p65 at Ser536, ab76302), p65 (ab32536), FLCN (ab124885), FNIP1 (ab215725), LAMP1 (ab278043), Flag-tag (ab125243), Myc-tag (ab206486), GST (ab19256), MBP (ab119994), histone H3 (ab5103), or β-actin (ab7291, Abcam Inc., Cambridge, MA). The specificity of commercial antibodies was validated by gene over-expression or silencing experiments, with isotype IgG as a negative control.

***Dual-luciferase reporter assay*:** Amplicons of human *NR1D1* (-1443/+100) or *RIOK3* (-2469/+801) promoter regions were generated from genomic DNA by PCR, and ligated into pGL3-Basic luciferase reporter vector (Promega). Site-directed mutagenesis of the RORB binding site was performed using Q5® Site-Directed Mutagenesis Kit (New England Biolabs, Inc) with mutation-specific primers (Table S4). For analyzing RORB transactivation, luciferase reporter constructs were generated through ligating oligonucleotide pairs with four typical RORB binding motifs (Table S4) into pGL4.1 vector (Promega). These constructs were transfected into tumor cells using Lipofectamine 3000 (Invitrogen), while transfection efficiencies were monitored by co-transfection of pNF-κB-Luc reporter plasmid (Agilent Technologies, Santa Clara, CA). The luciferase activity was measured by using LiveCell™ Dual-Luciferase Assay Kit (Biotium, Fremont, CA) and a GloMax® Luminescence Reader (Promega), while transcriptional activity was determined by Firefly/*Renila* activity ratio.

***Chromatin immunoprecipitation (ChIP) and ChIP-seq*:** ChIP assay was performed with the EZ-ChIP kit (Millipore, Burlington, MA), and antibodies against RORB (Abcam Inc., ab228650) or NF-κB p65

(ab218533). Two confluent 55 cm^2 dishes (1×10^7 cells per dish) were collected, while formaldehyde was added dropwise to the media (0.75% final concentration) for 10 minutes to cross-link proteins to DNA, with incubation under gentle rotation. Following treatment with glycine (125 $mmol\cdot L^{-1}$ final concentration) and two washes with 10 ml of ice-cold phosphate-buffered saline (PBS), cells were centrifuged to form pellets and then reconstituted in ChIP lysis buffer (Thermo Fisher Scientific, Inc., Waltham, MA; 750 μl per 1×10^7 cells). Chromatin fragmentation to 200-500 bp was achieved by sonicating lysates on ice using 20-second pulses. Sheared chromatin samples were then subjected to immunoprecipitation through overnight incubation with 3 μg of target-specific antibodies or control IgG. Captured immuno-complexes underwent sequential rinsing with ChIP wash solution (Thermo Fisher Scientific, Inc.) followed by elution in a buffer containing 1.0% sodium dodecyl sulfate (SDS) and 1.0 $mol\cdot L^{-1}$ $NaHCO_3$. Real-time quantitative PCR (qPCR) was utilized with SYBR Green PCR Master Mix (Takara) along with primers (Table S3). Isotype IgG was used as a negative control to standardize the immunoprecipitated DNA. For ChIP-seq, after library preparation, Illumina HiSeq X Ten was used for transcriptome sequencing (Wuhan SeqHealth Technology Co., Ltd., China). The 100-bp paired-end sequences were aligned to genomic features with HTSeq v0.6.0, and the number of transcript fragments (FPKM) per million mapped fragments was analyzed. The dataset was submitted to Gene Expression Omnibus (GEO), under accession GSE305134.

Gene over-expression or knockdown**:** The coding sequence (CDS) of human *RORB* (1370 bp) or *RBM10* (2793 bp) was amplified from NB specimens (Table S4), while their full-length fragments or truncations were inserted into pcDNA3.1 (Invitrogen), pCMV-3Tag-1A (Addgene, Cambridge, MA), pCMV-N-MYC (Addgene), pGEX-6P-1 (Beyotime Biotechnology, Haimen, China), pMAL-c4X (Addgene), or CV186 (Genechem Co., Ltd, Shanghai, China). Targeted mutations in *RBM10* and *RORB* were introduced via the GeneTailor™ mutagenesis kit (Invitrogen), employing primer pairs listed in Table S4. Lentiviral short hairpin RNA (shRNA) constructs were generated by inserting target-specific hairpin sequences (Table S5) into the GV298 backbone (Genechem Co.). After transfection via Lipofectamine 3000 (Invitrogen) or Genesilencer Transfection Reagent (Genlantis, San Diego, CA), stable transfectants were selected under continuous neomycin or prompycin (Thermo Fisher Scientific, Inc.), and validated by real-time qRT-PCR or western blot assay.

Gene expression restoration**:** The *RORB* expression vector was used to transfect tumor cells via Lipofectamine 3000 (Invitrogen) in order to prevent gene expression affected by *RBM10* over-expression. Using Genesilencer Transfection Reagent (Genlantis), shRNAs specific for *NR1D1* or *RIOK3* (Table S5) or expression vector of NF-κB *p65* (Genechem Co., Ltd) were transfected for rescuing target gene expression impacted by ectopic expression of *RORB*. The transfection efficiencies for restoring gene expression were validated by real-time qRT-PCR or western blot assay. As controls, an empty vector or scrambled shRNA (sh-Scb) was used (Table S5).

Lentivirus production**:** Lentivirus production was achieved by transfection of HEK-293T cells with transfer vector along with psPAX2 and pMD2G packaging plasmids (Addgene). Lentiviral particles were collected at 36 and 60 hours intervals following transfection, then clarified using low-protein-binding PVDF filters (0.45 μm pore size; Millipore). Lentiviral particles were pelleted by ultracentrifugation (120,000 × g, 2 hours, 4°C) and resuspended in 1/100 volume of PBS, achieving 100-fold concentration. The lentivirus was used to infect tumor cells within 48 hours, while transfection efficiencies were monitored by mCherry fluorescence observation.

Lysosome staining**:** For lysosomal visualization, cells were seeded on 24-well plates and treated with LysoTracker Red DND-99 (100 $nmol\cdot L^{-1}$; Sigma) in complete medium and maintained at physiological

temperature (37°C) for one hour prior to imaging. For nuclear visualization, cells were treated with Hoechst 33342 (2 $\mu g \cdot ml^{-1}$) in growth medium, followed by confocal microscopy (Olympus FV3000, Japan).

RNA sequencing (RNA-seq): Total RNA was extracted from 1×10^6 cells with RNeasy Mini Kit (Qiagen). RNA-seq libraries were prepared by Wuhan SeqHealth Technology Co., Ltd. following standard Illumina protocols, with subsequent sequencing on the HiSeq X Ten platform to generate 100 nucleotide paired-end reads. Gene-level read counts were quantified using HTSeq (version 0.6.0) with default union-counting mode, followed by FPKM normalization to account for transcript length and sequencing depth variations. All sequencing datasets were accessible through GEO database (GSE305133).

Co-IP and mass spectrometry: Co-IP assay was carried out by previously published methodology, with antibodies specific for RORB (ab228650), RBM10 (ab72423), Flag-tag (ab125243), or Myc-tag (ab206486, Abcam Inc.). Bead-bound proteins were released, separated using SDS-polyacrylamide gel electrophoresis (PAGE), and detected via Coomassie blue staining, immunoblotting, or proteomic profiling (Wuhan SpecAlly Life Technology Co., Ltd, China). The isotype IgG was used as a negative control.

GST pull-down: To generate truncated variants of *RORB* and *RBM10*, PCR amplification was undertaken with specific primers (Table S4). The amplified fragments were subsequently subcloned into pGEX-6P-1 or pMAL-c4X vector (Addgene), and then transformed into *E. coli* for generating GST-tagged RORB and MBP-tagged RBM10 proteins. The GST-tagged RORB was incubated with MBP-tagged RBM10 protein. Then, immunoprecipitation was performed with anti-GST or anti-MBP magnetic beads (Millipore), and associated proteins were analyzed by immunoblotting assay using antibodies specific for GST (ab19256) or MBP (ab119994, Abcam Inc.).

BiFC assay: The split-venus system was employed by cloning *RORB* (1370 bp) or *RBM10* (2793 bp) cDNA into pBiFC vectors (Addgene), followed by dual transfection into tumor cells (Lipofectamine 2000, Invitrogen) for 24 hours. Following transfection, cells were incubated under standard culture conditions (37°C, 5% CO_2) for 10 hours to facilitate fluorophore maturation. Venus reconstitution was subsequently visualized via confocal microscopy (excitation: 488 nm; emission: 500-550 nm).

In vitro phase separation assay: Recombinant His-tagged proteins were generated by transforming *RORB*/*RBM10* constructs into BL21 *E. coli* (Thermo Fisher Scientific, Inc.), then isolating the proteins through His-tag Affinity Purification Kit (Thermo Fisher Scientific, Inc.). SDS-PAGE and Coomassie blue staining were applied for detecting recombinant proteins, while ImageJ (https://imagej.nih.gov/ij) was employed for densitometric purity assessment. Phase separation assays were conducted using glass-bottomed dishes. For droplet formation assay, 40 $\mu mol \cdot L^{-1}$ protein solutions in buffer (containing 10% glycerol, 1.0 $mmol \cdot L^{-1}$ dithiothreitol, and 50 $mmol \cdot L^{-1}$ Tris-HCl at pH 7.5) were mixed with 10% PEG-8000 and imaged with an oil-immersion Olympus FV3000 confocal system.

Phase separation in live cells: Cells expressing mCherry-fused proteins or stained for endogenous targets were cultured on glass-bottom dishes (MatTek, Ashland, MA), with nuclear counterstained by 4',6-diamidino-2-phenylindole (DAPI, Thermo Fisher Scientific, Inc., 10 minutes). Confocal microscopy (Olympus FV3000) was performed after PBS washes. Phase separation puncta (>0.5 μm diameter) were analyzed.

Fluorescence recovery after photobleaching (FRAP): *In vitro* FRAP experiments were performed with

an Olympus FV3000 confocal laser scanning microscope. Target droplets were photobleached for 10 seconds using alternating 488 nm and 561 nm laser pulses at 50% maximum power, with simultaneous time-lapse imaging (1 frame/second) to monitor fluorescence recovery. All imaging was conducted under controlled environmental conditions (37°C, 5% CO_2). FRAP assays were conducted in live cells using a climate-controlled chamber (37°C, 5% CO_2). Target regions were bleached (488/561 nm lasers, 50% power, 5 seconds) and imaged at 500 ms intervals for 60 seconds (Olympus FV3000, 60×oil objective). Utilizing the FIJI/ImageJ (https://imagej.nih.gov/ij) software, the intensities of fluorescence were compared to pre-bleach values after background correction.

***Inhibitory peptide synthesis*:** Structure-guided inhibitory peptides targeting RBM10-RORB interaction interface were designed. Initial structural models of RBM10 and RORB were generated *in silico* using the Phyre2 web server (https://www.sbg.bio.ic.ac.uk/phyre2), producing corresponding PDB files. The interaction between these proteins was then predicted via molecular docking with the ZDOCK program (https://zdock.umassmed.edu). The resulting docked complex (PDB file) was subsequently analyzed using the Peptiderive server (https://rosie.rosettacommons.org/peptiderive), configured to derive peptides precisely 12 amino acids in length. Peptides exhibiting the highest predicted binding affinity (docking score) alongside their corresponding protein interaction domains were identified. The HIV-1 TAT peptide (YGRKKRRQRRR) was incorporated as a cell-penetrating motif. Therefore, peptides were synthesized (Genscript, Nanjing, China) with dual modifications: N-terminal biotin-TAT (biotin-YGRKKRRQRRR) for affinity purification and C-terminal fluorescein isothiocyanate (FITC) for fluorescence detection, and purified with more than 95% homogeneity.

***Biotinylated peptide pull-down*:** Following extraction with 1× RIPA lysis buffer (Promega), cellular proteins were treated with biotinylated peptide at 4°C for 16 hours. Cell lysates were incubated with streptavidin-agarose beads (Thermo Fisher Scientific, Inc.) at 4°C for 2 hours. After extensive washing, the bound proteins were detected by immunoblotting.

***CTSD activity assay*:** CTSD enzymatic activity was quantified using a commercial Cathepsin D Activity Assay Kit (ab65302, Abcam Inc.). Briefly, tumor cells (1×10^6) was collected and treated with Reaction Mix at 37ºC for 1-2 hours. The microplate reader was applied for fluorometric assay at the wavelength of 328/460 nm.

***Cellular viability assay*:** Cellular viability was assessed by 3-(4,5-Dimethylthiazol-2-yl)-2,5-diphenyltetrazolium bromide (MTT, Sigma) colorimetry. Briefly, viable tumor cells (3×10^3 per well) were seeded in 96-well plates and exposed to MTT (5 mg/ml). Following a 4-hour incubation at 37°C, the liquid phase was aspirated. Formazan deposits were solubilized in 150 µl dimethylsulfoxide (Sigma), and optical density of dissolved formazan was recorded at 570 nm versus 630 nm reference.

***Immunofluorescence assay*:** Cells grown on glass coverslips underwent blocking with 10% goat serum (37°C, 1 hour) prior to incubation for 2 hours with primary antibodies against either GAP43 (Abcam ab75810; 1:100 dilution) or NF-200 (Abcam ab215903; 1:100 dilution). Following treatment with Alexa Fluor 594-conjugated goat anti-rabbit IgG (Abcam ab150160), nuclei were counterstained with 300 $nmol\cdot L^{-1}$ DAPI.

***Cell cycle and apoptosis assays*:** Cell cycle distribution and apoptotic status were assessed using flow cytometry. For cell cycle analysis, tumor cells underwent fixation in 70% ice-cold ethanol followed by treatment with RNase A (2 mg/ml; Sigma). DNA was subsequently stained with propidium iodide (PI, 20 mg/ml; Sigma) for 20 minutes prior to analysis on a BD Biosciences flow cytometer (San Jose, CA).

Apoptosis evaluation involved dual staining of tumor cells (5×10^5) with Annexin V-FITC and PI (Sigma) under light-protected conditions for 15 minutes, with fluorescence measurements acquired at 488 nm excitation using a BD Biosciences instrument (San Jose, CA).

Soft agar colony formation assay: Following preparation in 0.05% Noble agar (Thermo Fisher Scientific, Inc.) and complete medium, tumor cells (5×10^3) were plated on 6-well plates pre-coated with a 0.1% agar base layer. After 21 days of incubation, colonies were stained with 0.5 $mg\cdot ml^{-1}$ MTT (Sigma) at 37°C for 4 hours. Viable colonies (>50 μm diameter) were quantified under a microscope.

Cell invasion assay: Cells (1×10^5 in 200 μl serum-free medium) were seeded onto Matrigel-coated (1:8 dilution) upper chambers, while the lower chambers contained 600 μl of chemoattractant medium containing 10% FBS. Chambers were incubated for 24 hours at 37°C under 5% CO_2 humidified atmosphere. Cells that invaded to the lower membrane surface were fixed with 4% paraformaldehyde for 15 minutes at room temperature, and subsequently stained with 0.1% crystal violet solution (Sigma) for 20 minutes. Membranes were excised and mounted on slides for brightfield microscopy. Quantification of invaded cells was carried out using established evaluation standards.

Nude mice studies: Animal experiments followed NIH guidelines and were approved by Experimental Animal Ethics at Huazhong University of Science and Technology (Ethical Approval No. 2017-3208). For *in vivo* tumorigenesis assays, four-week-old BALB/c nude mice (n=5 per group) were randomly allocated and received subcutaneous inoculation of 1×10^6 tumor cells (in 100 μl PBS) into the dorsal flank. After a period of one month, mice were euthanized to assess tumor mass. For experimental metastasis assays, four-week-old BALB/c nude mice (n=5 per group) were randomly assigned and injected intravenously via the lateral tail vein with 0.4×10^6 tumor cells suspended in 100 μl PBS. Mice were euthanized when they exhibited signs of illness or paralysis. For *in vivo* treatment evaluation, BALB/c nude mice were treated with injections of tumor cells (1×10^6 into dorsal flank or 0.4×10^6 via tail vein). One week post-inoculation, mice were randomly allocated to receive cell-penetrating peptide (Genscript) administered either intratumorally or intravenously. Tumor volumes and survival of mice were recorded. *In vivo* imaging was conducted weekly with VIS® Lumina III (PerkinElmer, Waltham, MA).

Patient specimens: This study involving human tissues was approved by the Institutional Review Board of Union Hospital, Tongji Medical College (Ethical Approval No. 2023-0519). All procedures were performed in accordance with the ethical standards of the Declaration of Helsinki. For all pediatric participants, written informed consent was secured from legal guardians. NB tissues were collected during surgical resections at Union Hospital, Tongji Medical College, excluding patients with prior chemotherapy/radiotherapy. Normal dorsal root ganglia used as controls were sourced from therapeutic abortions. All tissue specimens were immediately snap-frozen in liquid nitrogen following collection, histologically verified by a certified pathologist, and maintained at -80°C until processing.

Immunohistochemical staining: Immunohistochemical staining was carried out using standard protocols and following antibodies: anti-Ki-67 (ab92742; 1:100), anti-RORB (ab188756, 1:200), or anti-RBM10 (ab220847, Abcam Inc.; 1:200). Specificity was verified using antigen-blocking peptides or IgG isotype control. The intensity and positive cell percentage of staining reaction was evaluated by two pathologists blind to group assignment.

Statistical analysis: The data were analyzed by GraphPad 8.0 software (GraphPad Software, Boston, MA). All quantitative findings were expressed as mean ± standard error of the mean (SEM). Gene expression cutoff values were established based on the median expression levels. To quantify the significant

concordance of independent gene sets, Fisher's exact test was implemented. To evaluate differences among tumor cells or tissues, statistical comparisons were performed using either non-parametric Mann-Whitney U test (for non-normal distributions), one-way analysis of variance (ANOVA, for multiple distributed parametric data), or Student's *t*-test (for normally distributed parametric data). Kaplan-Meier curves were applied for analyzing survival data, while group comparisons were performed using two-sided log-rank test. Statistical significance was assessed by using two-tailed tests,, with significance defined as false discovery rate (FDR)-adjusted *P*-value less than 0.05.
